# Bioleaching for critical metal recovery from bauxite residue- unlocking waste valorization

**DOI:** 10.3389/fbioe.2025.1685819

**Published:** 2025-11-26

**Authors:** Fernanda Soto-Montandon, Rosemary Gillane, Esteban Marcellin, Susan T. L. Harrison, Denys Villa-Gomez

**Affiliations:** 1 Australian Institute for Bioengineering and Nanotechnology, University of Queensland, Brisbane, QLD, Australia; 2 School of Civil Engineering, University of Queensland, Brisbane, QLD, Australia; 3 Faculty of Engineering, Architecture and Information Technology, University of Queensland, Brisbane, QLD, Australia

**Keywords:** bioleaching, bauxite residue, waste valorization, critical minerals, circular economy

## Abstract

The increasing problem of waste from alumina refineries endangers ecosystems and local communities. With over 4 billion tons of bauxite residue stored globally, more than 140 million tons generated annually, and less than 3% currently reused, the situation is unsustainable. However, bauxite residue also contains critical metals essential for advancing sustainable technologies, aligning with the United Nations’ 7^th^ Sustainable Development Goal (SDG). Fully recovering these valuable elements and reusing the waste not only addresses environmental concerns but also supports a resilient and sustainable supply of materials needed for the green energy transition. Given the environmental drawbacks of traditional extraction methods, biotechnological approaches show promise as an environmentally responsible and cost-effective alternative, reinforcing circular economy principles and supporting the 12^th^ SDG, which promotes responsible resource use and the reduction of hazardous waste. This review offers a novel, integrated evaluation of bauxite residue valorization, combining an overview of its composition and characteristics with a detailed examination of bioleaching-based recovery of rare earth elements, gallium, vanadium, and titanium. Special emphasis is placed on selecting optimal microorganisms, understanding the metabolic pathways behind bioleaching agent production, and refining strategies to enhance process efficiency and microbial performance. Additionally, it highlights how circular economy approaches can drive resource-efficient and sustainable utilization of alkaline residues, providing a perspective not covered in previous studies.

## 1 Introduction

Bauxite residue is one of the most abundant industrial by-products generated globally. It is produced from the alumina (Al_2_O_3_) extraction from bauxite ore (Kvande, 2015; Healy, 2022). For every ton of alumina extracted, between one and two tons of this highly alkaline residue are generated, resulting in a staggering global inventory of approximately 4 billion tons, with an additional 140 to 150 million tons accumulating each year (Kumar et al., 2006; Cablik, 2007). Projections suggest that, unless transformative recovery and utilization practices are implemented, global bauxite residue stockpiles are projected to increase by more than twofold to 9–10 billion tons by 2050, driven by continued growth in aluminum demand (Aung et al., 2021; Healy, 2022; Yi et al., 2024).

Despite the scale of production, less than 3% of bauxite residue is currently reused (U. S. Environmental Protection Agency, 2024), leaving the vast majority stored in tailings facilities, which pose long-term environmental hazards, including soil and groundwater contamination, air pollution, and substantial land occupation. This *status quo* is increasingly at odds with the United Nations Sustainable Development Goals (SDGs), particularly the 12th SDG, which calls for responsible consumption, improved resource efficiency, and minimization of waste generation (United Nations General Assembly, 2015).

**FIGURE 1:**
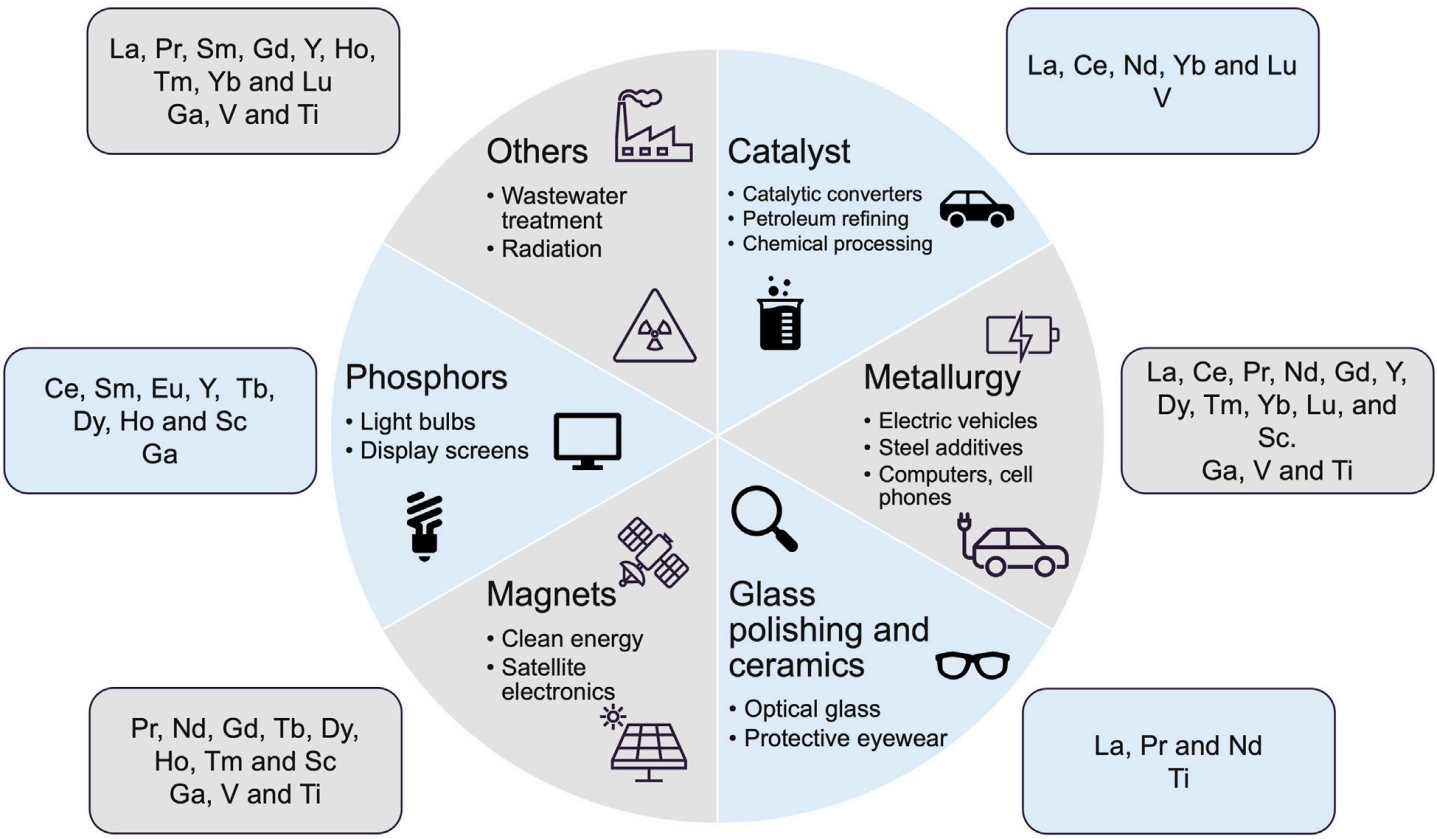
Principal applications of REEs, Ga (Butcher and Brown, 2014; Foley et al., 2017), V and Ti (Suresh et al., 2023). REEs include lanthanum (La) (Aaseth and Berlinger, 2022), cerium (Ce) (Gad, 2024), praseodymium (Pr), neodymium (Nd), promethium (Pm), samarium (Sm), europium (Eu), gadolinium (Gd), terbium (Tb), dysprosium (Dy), holmium (Ho), erbium (Er), thulium (Tm), ytterbium (Yb), lutetium (Lu), Sc and yttrium (Y) (Šulc and Jelínková, 2013; Smith Stegen, 2015; Chernoburova and Chagnes, 2023; Dürig et al., 2023; U.S. Geological Survey, 2025).

At the same time, bauxite residue presents a valuable, yet underutilized, opportunity for resource recovery. It contains considerable concentrations of critical metals, including scandium (Sc), rare earth elements (REEs), gallium (Ga), vanadium (V), and titanium (Ti), which are essential for a wide range of modern technologies, especially those tied to clean energy, digitalization, and defense applications (Figure 1). These metals are defined as “critical” due to their strategic importance and the risk of supply disruption from geopolitical tensions, limited reserves, or trade constraints (European Commission Directorate-General for Internal Market, Industry, Entrepreneurship SMEs, 2023; Geoscience Australia, 2024b; Survey, 2024). Recovering these elements from the alumina refinery residue not only represents a way to mitigate environmental hazards but also constitutes a strategic opportunity to strengthen supply chain resilience and support global green transition goals, particularly those aligned with the 7^th^ SDG on clean energy (United Nations General Assembly, 2015).

In recent years, the circular economy framework has gained prominence as a guiding principle in sustainable materials management, emphasizing the continuous reuse and recovery of resources across product life cycles (De Jesus and Mendonça, 2018; Kalmykova et al., 2018). Such approaches have been explored across both organic and inorganic material flows with examples including the wastewater biorefinery approach (Pott et al., 2018) and the valorization of mining wastes (Harrison et al., 2020; Villa Gomez et al., 2024). Within this framework, bauxite residue valorization has focused on two main pathways: 1) value-added utilization, which includes incorporation into construction materials, use in environmental remediation, catalytic applications, and its transformation to technosol; and 2) resource recovery, aimed at extracting critical metals. While considerable progress has been made in value added utilization pathways, some of which are already in commercial or near-commercial use, the potential for environmentally sustainable recovery of critical metals from bauxite residue remains largely underexplored (Kehagia, 2014; Santini and Fey, 2016; Kriskova et al., 2025). Currently, there are no technically and economically feasible processing routes, and the technology readiness levels remain low, particularly for elements beyond the conventional extraction of iron (Fe), aluminum (Al), and Ti, which dominate existing industrial-scale recovery efforts (Balomnenos et al., 2016; Vielma et al., 2025).

Conventional extraction methods are often hindered by severe economic and environmental constraints. They typically rely on high energy inputs, elevated temperatures and pressures, and aggressive chemical reagents, resulting in high costs, complex infrastructure, and secondary environmental impacts such as acid waste streams or alkaline effluents (Gao et al., 2020; Zapp et al., 2022; Yıldız et al., 2024). These limitations have sparked interest in alternative, low-impact technologies that can extract critical metals more sustainably. Among them, bioleaching, the microbiologically driven solubilization/complexation of metals, has emerged as a promising avenue. Bioleaching offers the potential for lower energy use, milder operational conditions, and substantially reduced reliance on harsh chemicals. Despite its proven success in recovering metals from other industrial waste streams and ores, bioleaching remains in the early stages of development for bauxite residue. Several fundamental challenges remain unresolved, including slow kinetics, low metal selectivity, limited microorganism compatibility with the highly alkaline matrix, and a need for process optimization at larger scales.

Understanding bioleaching mechanisms is fundamental to advancing the sustainable recovery of critical metals from bauxite residue. While previous reviews have explored the potential of bauxite residue as a secondary source of valuable metals and discussed strategies for their recovery and process optimization (Swain et al., 2022; Harmaji et al., 2024; Fang et al., 2025; Padhan and Paul, 2025), a comprehensive synthesis that integrates bioleaching techniques, microbial selection criteria, and the underlying mechanisms of metal complexation, alongside an evaluation of the metabolic pathways of bioleaching organisms, remains limited. This review seeks to address these gaps by integrating current knowledge on bioleaching methods and microbial metabolism, emphasizing approaches to enhance extraction efficiency through strategies such as optimizing leaching parameters, improving microbial tolerance, and increasing organic acid production, all of which considerably influence process performance. We critically assess the current state of research on the bioleaching of bauxite residue, focusing on its applicability for the recovery of critical metals. The review provides an overview of residue composition and its relevance to circular economy frameworks, summarizes recent advances in bioleaching mechanisms and microbial processes, and identifies key technological developments and existing knowledge gaps that must be addressed to establish bioleaching as a viable industrial solution. Through this lens, we aim to pave the way for future research and development in transforming bauxite residue from an environmental liability into a valuable resource stream that fits within a sustainable and circular materials economy.

## 2 Bauxite residue characteristics and critical metals recovery potential

Bauxite residue is a highly alkaline by-product generated predominantly during the Bayer process, which accounts for over 95% of global alumina production (Agrawal and Dhawan, 2021). Its chemical composition varies depending on the processing method and ore type, but typically includes oxides of Fe, Al, Ti, calcium (Ca), and sodium (Na) (Gräfe et al., 2011b). In Bayer-derived bauxite residue, Fe_2_O_3_ and Al_2_O_3_ often dominates, while sintering-derived residues, more common in China, are richer in silica and lime (Table 1) (Sun et al., 2019).

**TABLE 1:** Chemical composition of bauxite residue samples worldwide. Values expressed as weight percentage (by oxides), obtained via XRF analysis.

Country	Refinery	Processing	Fe_2_O_3_	Al_2_O_3_	TiO_2_	SiO_2_	Na_2_O	CaO	References
Australia	Kwinana	Bayer	28.5	24.0	3.1	18.8	3.4	5.3	Snars and Gilkes (2009)
Australia	Wagerup	Bayer	29.6	17.3	2.7	30.0	3.2	3.6	Snars and Gilkes (2009)
Australia	Worsley	Bayer	56.9	15.6	4.5	3.0	2.2	2.4	Snars and Gilkes (2009)
Australia	Queensland Alumina Limited	Bayer	30.7	18.6	7.0	16.0	8.6	2.5	Snars and Gilkes (2009)
Australia	Yarwun	Bayer	34.5	22.5	6.8	13.9	9.4	1.8	Liu et al. (2016)
China	Henan	Bayer	16.7	23.3	5.2	20.4	7.4	11.4	Liu et al. (2017)
China	Guizhou	Bayer	26.4	18.9	7.4	8.5	4.8	21.8	Wang and Liu (2012)
China	Guizhou	Sintering	8.0	10.4	7.1	17.3	3.5	40.2	Wang and Liu (2012)
China	Shandong	Sintering	5.7	8.3	-	32.5	2.3	41.6	Liu et al. (2014)
China	Shanxi	Sintering	6.8	10.5	2.6	22.2	3.0	42.3	Liu et al. (2014)
Greece	Alumine de Greece	Bayer	42.5	15.6	5.9	9.2	2.4	19.7	Ochsenkühn-Petropulu et al. (1996)
Hungary	Ajka	Bayer	42.1	14.8	5.2	13.5	8.9	6.1	Prasad and Singh (1997)
India	Damanjodi	Bayer	53.0	15.5	5.0	7.1	4.4	2.1	Hena et al. (2022)
India	Hindalco	Bayer	35.5	16.8	7.7	15.6	13.6	1.2	Alam et al. (2018)
Brazil	Maranhão	Bayer	38.3	22.1	3.5	12.2	5.8	2.6	Silveira et al. (2025)
Brazil	São Paulo	Bayer	31.8	21.7	3.6	16.5	7.1	4.3	Silveira et al. (2025)

The mineral composition of bauxite residue varies considerably depending on the processing method and the specific parameters used. The major mineral phases in Bayer-derived bauxite residue include hematite, goethite, quartz, gibbsite, boehmite, anatase, sodium aluminosilicates (e.g., sodalite and cancrinite), and calcite, with minor occurrences of muscovite, feldspar, rutile, gypsum, tricalcium aluminate, and halite (Snars and Gilkes, 2009). In contrast, in sintering-derived residue, the dominant phases are wollastonite, limonite, calcite, anorthite, sodium aluminosilicate, brownmillerite, and perovskite (Liu et al., 2009b). Wang and Liu (2012) reported that, in samples from the same alumina refinery, the main phases in Bayer-derived residue are perovskite, hematite, sodium aluminate, calcite, and aragonite, whereas in sintering-derived residue, they include dicalcium silicate, calcite, perovskite, and magnetite. However, the specific phase abundances were not provided.

The strong alkalinity (pH 10.5–12.8) and elevated salinity (ESP 53%–91%) (Jiang et al., 2023), combined with the presence of toxic metals (e.g., arsenic, cadmium, chromium, nickel, lead) (Burke et al., 2012; Higgins et al., 2016; Zhang et al., 2022) and naturally occurring radionuclides (e.g., radium-226, thorium-232), pose considerable challenges for long-term storage and environmental safety (Goronovski et al., 2021). Most bauxite residue is currently managed through dry stacking systems, which helps to reduce the land use and remove the highly alkaline liquid attached to the residue in the form of OH^−^, CO_3_^2-^ and Al(OH)_4_^-^. However, more than 75% of the waste’s alkalinity is present in the form of insoluble compounds such as sodalite, cancrinite, hydrogarnet, calcite, tricalcium aluminate, sodium carbonate, sodium aluminate, sodium bicarbonate, and sodium silicate (Xue et al., 2016; Lyu et al., 2021).

Alternative treatment strategies such as seawater neutralization and CO_2_ carbonation are applied to dissolve the mineral phases and react with the alkaline products to reduce the alkalinity (Rai et al., 2017; Kannan et al., 2021). Though, these approaches have limitations, including high water requirements (Yang J. et al., 2025) and risks of pH rebound due to residual alkaline minerals (Rivera et al., 2017; Harmaji et al., 2024). This complex environmental context necessitates the development of integrated strategies that not only mitigate risks but also valorize the residue as a resource.

**FIGURE 2:**
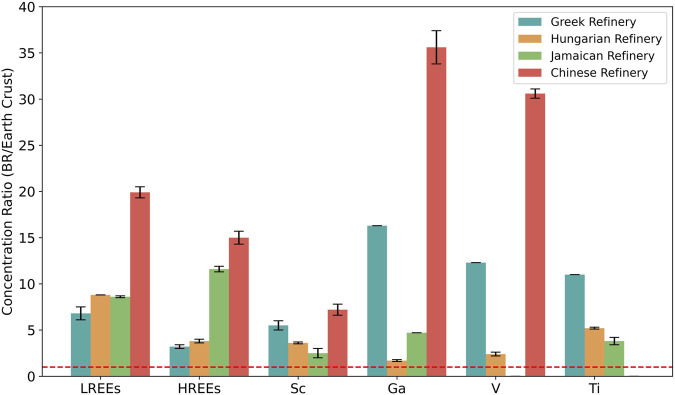
Average concentration ratios of LREEs, HREEs, Sc, Ga, V, and Ti in Greek refinery (Borra et al., 2015; Toli et al., 2023), Hungarian refinery (Ujaczki et al., 2017), Jamaican refinery (Patterson et al., 1986; Narayanan et al., 2019), and Chinese refinery (Qu and Lian, 2013) produced bauxite residues, and in the Earth’s crust (Thomas, 2018; Geoscience Australia, 2024a), with standard deviations shown in the graph (see Supplementary Material). The dashed line represents the point at which the critical metal concentration in bauxite residue (BR) equals that in the Earth’s crust (a ratio of 1).

Importantly, bauxite residue is enriched with a range of critical metals whose concentrations often exceed their natural crustal abundance by multiple folds. For instance, bauxite residue from a Greek refinery contains these metals at concentrations ranging from 3 to 16.3 times higher than crustal averages (Figure 2). In Hungarian bauxite residue, concentrations range from 1.6 to 8.8 times higher, with a higher proportion of light rare earth elements (LREEs) compared to heavy rare earth elements (HREEs). In contrast, Jamaican bauxite residue displays the opposite trend, with HREEs enriched by a factor of over 11.3 and LREEs by 8.5. Greek bauxite residue contains a high concentration of Ga, V, and Ti, with enrichment factors exceeding 10. Chinese bauxite residue shows a similar trend to Hungarian bauxite residue, with higher concentrations of LREEs compared to HREEs. This residue also exhibits a considerable enrichment factor for Ga and V (Figure 2). These enrichment patterns underscore the substantial, yet underutilized, resource potential of bauxite residue for multiple critical metals.

REEs are a group of 17 chemically similar elements, including the 15 lanthanides, plus Sc and Y, due to their similar properties and co-occurrence in ores. They are typically categorized into light REEs (LREEs: La to Gd) and heavy REEs (HREEs: Tb to Lu, including Y), while Sc is often treated separately due to its distinct characteristics (Balaram, 2019).

In bauxite residue, REEs largely mirror their mineral associations of the original bauxite ore, occurring within mineral phases such as cerianite (CeO_2_), fluorocarbonates such as synchysite [Ca(REE)(CO_3_)_2_F] and bastnäsite [Ce(CO_3_)F], hydroxylbastnäsite [REE(CO_3_)(OH)], xenotime (YPO_4_), monazite [(REE)PO_4_], or adsorbed onto the surfaces of clays and diaspore (Li et al., 2013; Vind et al., 2018b; Luo et al., 2023). They might also be adsorbed onto mineral surfaces in perovskite form or substitute for chemically similar ions within the mineral matrix (Borra et al., 2015; Gamaletsos et al., 2016; Vind et al., 2018b), for example, Sc which is commonly hosted in hematite and goethite (Vind et al., 2018c). In general, Sc accounts for more than 90% of the total REEs content in bauxite residue, and approximately 95% of the overall economic value of the REEs present (Binnemans et al., 2015; Rivera et al., 2017; Rivera et al., 2019). Nearly all REEs present in bauxite ore are transferred to the bauxite residue during alumina extraction, resulting in an enrichment factor of at least two (Figure 2) (Vind et al., 2018a). Ga is another critical metal of strategic importance associated with bauxite processing (Foley et al., 2017). Approximately 90% of the global Ga annual production is obtained as by-product of the alumina production (Yuxin et al., 2025). Due to its geochemical similarity to Al, approximately 70% of the Ga in bauxite is leached and retained in the Bayer liquor, while the remaining 30% is lost to bauxite residue in the form of Ga oxyhydroxide (α-GaOOH) and Ga hydroxide (Ga(OH)_3_) (Lu et al., 2017; Vind et al., 2018a).

V is also enriched in bauxite residue through its incorporation into mineral phases formed during the Bayer process. It is known that V in bauxite residue can replace hydroxyl groups in the tricalcium aluminate hydrates and calcium aluminum silicates (Burke et al., 2012; Smith, 2017; Vind et al., 2018a), and can also associate with Fe oxides and Ti-Fe minerals (Gräfe et al., 2011a). Similarly, Ti, another abundant constituent of bauxite residue, is typically present as rutile (TiO_2_), anatase (TiO_2_), perovskite (CaTiO_3_), or ilmenite (FeTiO_3_). The relative abundance of these phases depends on the original bauxite ore composition and the specific processing conditions used (Murty et al., 2023; Stopić et al., 2023).

## 3 Bioleaching of bauxite residue for critical metal recovery

Conventional recovery of critical metals from bauxite residue is characterized by the need for elevated temperatures and pressures, substantial initial capital investment, and/or the use of concentrated mineral acids or highly alkaline conditions, which can lead to excessive water consumption and environmental pollution (Fang et al., 2025; Tanvar and Mishra, 2025). These limitations have reinforced the need to develop more economically viable, sustainable, and environmentally responsible technologies. Bioleaching has emerged as a promising solution, employing microorganisms to convert target metals from their insoluble form in the bauxite residue matrix into a soluble form that can be easily separated.

### 3.1 Bioleaching configuration

**FIGURE 3:**
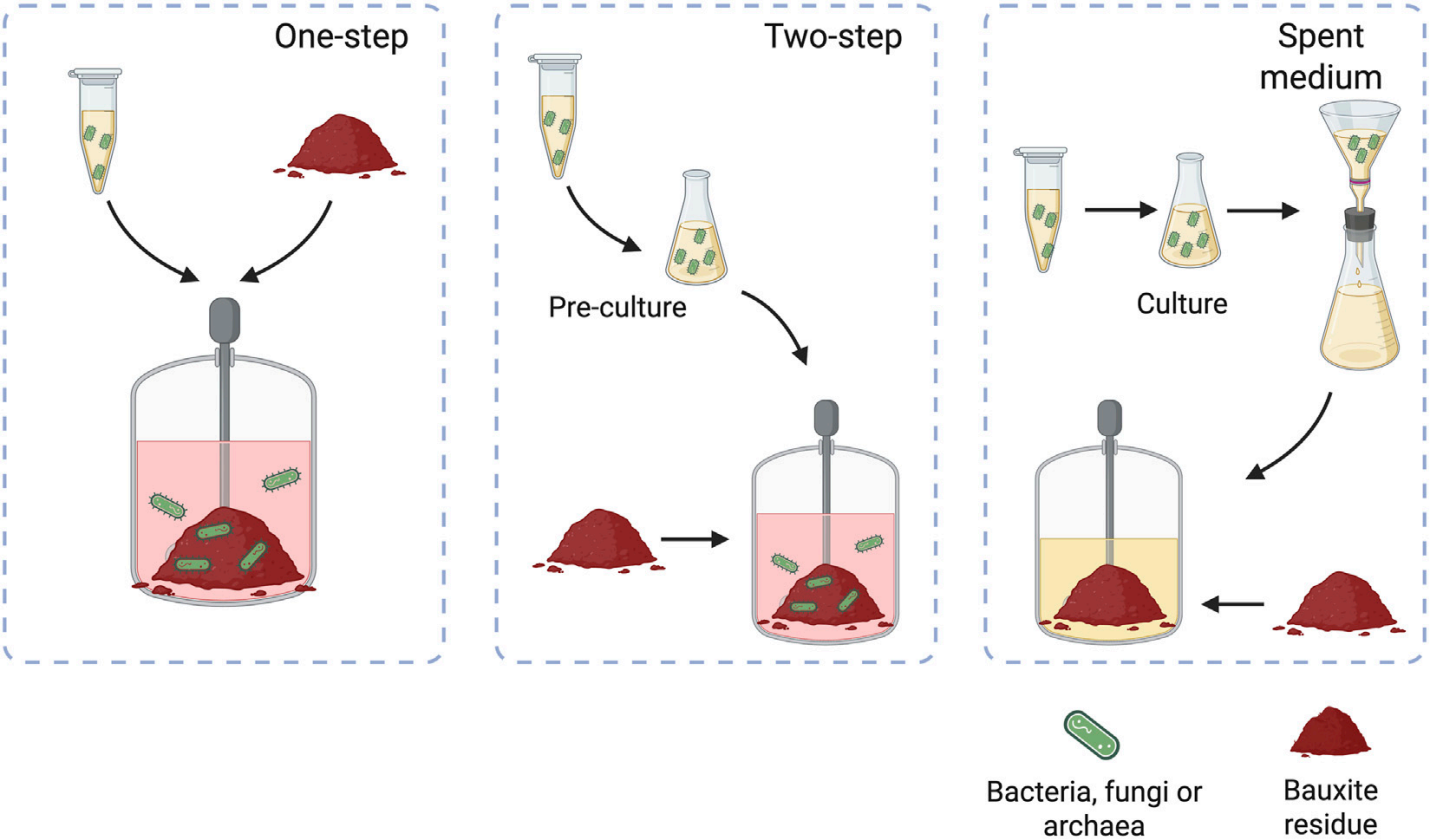
Bioleaching configurations when applied to bauxite residue. Adapted from (Yang et al., 2008a; Ren et al., 2009).

Bioleaching is typically implemented using one of three main configurations: one-step, two-step, or spent-medium systems (Figure 3), with the first two considered contact leaching because the microorganisms physically interact with the mineral surface, and the latter classified as contactless (Crundwell, 2003). In the one-step process, the microorganisms are directly inoculated into the medium containing the bauxite residue, allowing for simultaneous microbial growth and bioleaching. It has been reported that, although this approach often results in longer lag phases due to metal toxicity or pH stress, it can achieve high overall recoveries (Biswal and Balasubramanian, 2023).

Alternatively, the two-step method involves allowing the microbes to grow separately in a nutrient medium until they reach optimal density before introducing the bauxite residue. This configuration helps reduce microbial stress and minimizes lag phases, leading to more efficient metal solubilization under controlled conditions. The spent medium approach bypasses direct microbial exposure to bauxite residue altogether by using only the leachate (metabolite-rich medium) produced by the microorganisms. This strategy is particularly useful when dealing with toxic substrates or when employing genetically modified strains that require biocontainment.

### 3.2 Bioleaching mechanisms

**FIGURE 4:**
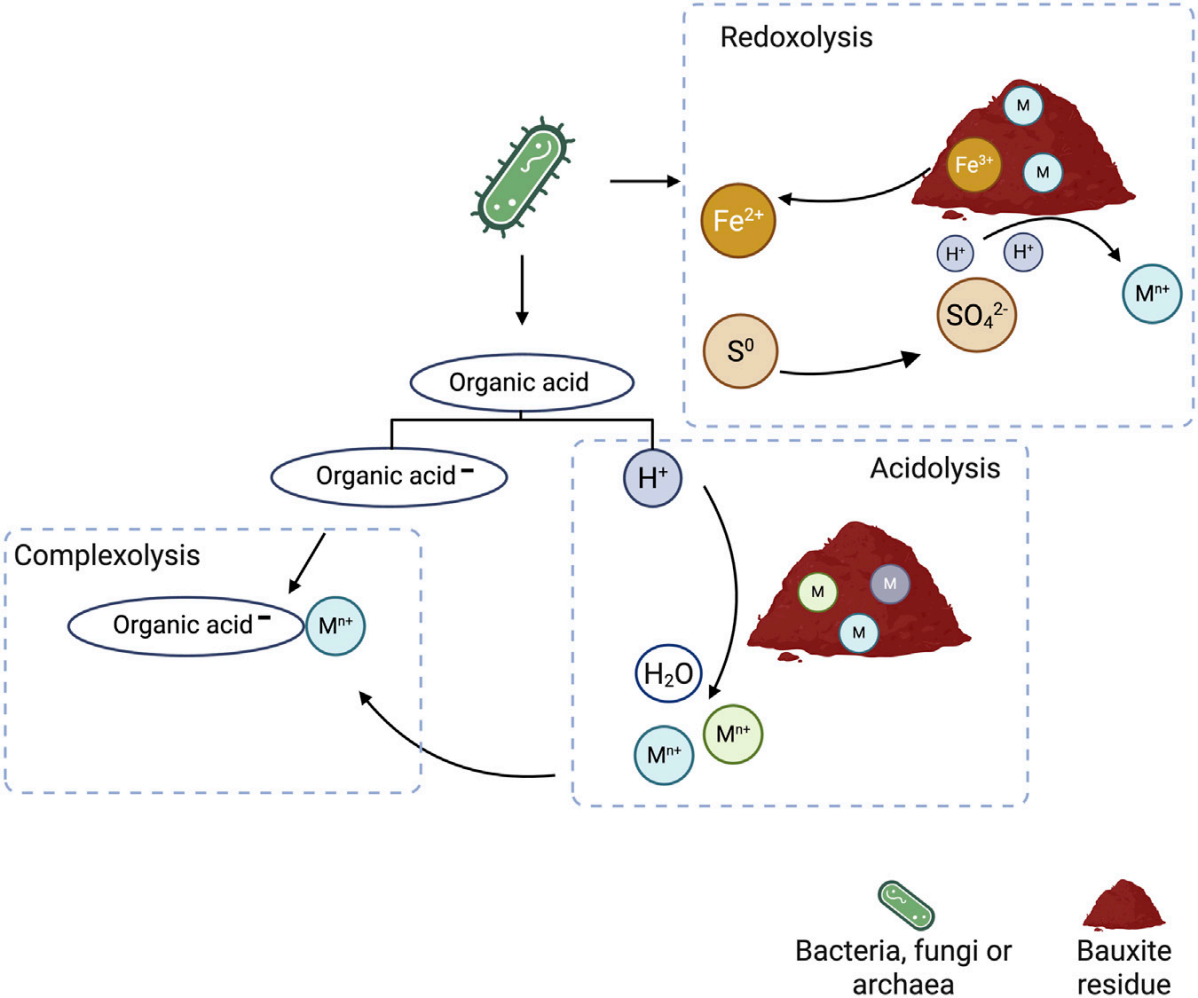
Bioleaching mechanisms in fungi, bacteria and other organisms. Iron reduction and sulfur oxidation were employed to elucidate the coupled redoxolysis and acidolysis mechanisms. An organic acid was used as a bioleaching agent to describe the acidolysis and complexolysis mechanisms. Adapted from (Zhang et al., 2020; Ilyas et al., 2021; Dong et al., 2023).

The bioleaching mechanisms refer to how the solubilization of metals by microorganisms, can occur via acidolysis, complexolysis, and redoxolysis. These mechanisms can act individually or in combination during bioleaching of bauxite residue (Figure 4). In acidolysis, microorganisms secrete organic or inorganic acids that lower the pH and mobilize metal ions through proton displacement from mineral structures. Studies have shown that the bioleaching rate of metals increases with the proton donor capacity of the acid. Similarly, higher acid concentrations positively impact the leaching rate and extent (Ilyas et al., 2021).

Complexolysis involves metal complexation with the organic acid through chelation, displacing the equilibrium, and enhancing the continuous dissolution of the metal ions. Even though complexolysis is slower than acidolysis, under less acidic conditions this mechanism controls the leaching rate (Dong et al., 2023). It has also been previously indicated that the concentration of the conjugate base, rather than hydronium ion availability, plays a critical role in determining the leaching efficiency of bauxite residue (Van Wyk et al., 2024).

In redoxolysis, the organisms enhance metal solubilization through electron transfer between the metal source and the microbes. This mechanism can be employed to recover metals from low-grade ores or secondary sources, such as electronic waste, or to decrease the toxicity of metal ions by reducing or oxidizing them into non-toxic or less toxic forms (Pathak et al., 2021). In bauxite residue, *Acidianus manzaensis* has been reported to release REEs from the solid matrix through redoxolysis and acidolysis in a two-stage bioleaching process. First, pyrite is oxidized under aerobic conditions to generate ferric iron and H_2_SO_4_. Subsequently, ferric iron is reduced to ferrous iron under anaerobic conditions when electron donors, such as elemental sulfur, are supplied, promoting metal solubilization by destabilizing ferric phases through redoxolysis (Zhang et al., 2020).

### 3.3 Bioleaching agents

The biological metal recovery from bauxite residue occurs through the secretion of metabolites to the extracellular media which interact closely with mineral surfaces to disrupt bonds and mobilize metal ions. Among these metabolites, low-molecular-weight organic acids are considered the main leaching agents. These organic acids, including citric, oxalic, tartaric, malic, gluconic, and acetic acid, are secreted by various bacterial and fungal species and play a pivotal role in acidolysis and complexolysis (Gadd, 1994).

**FIGURE 5:**
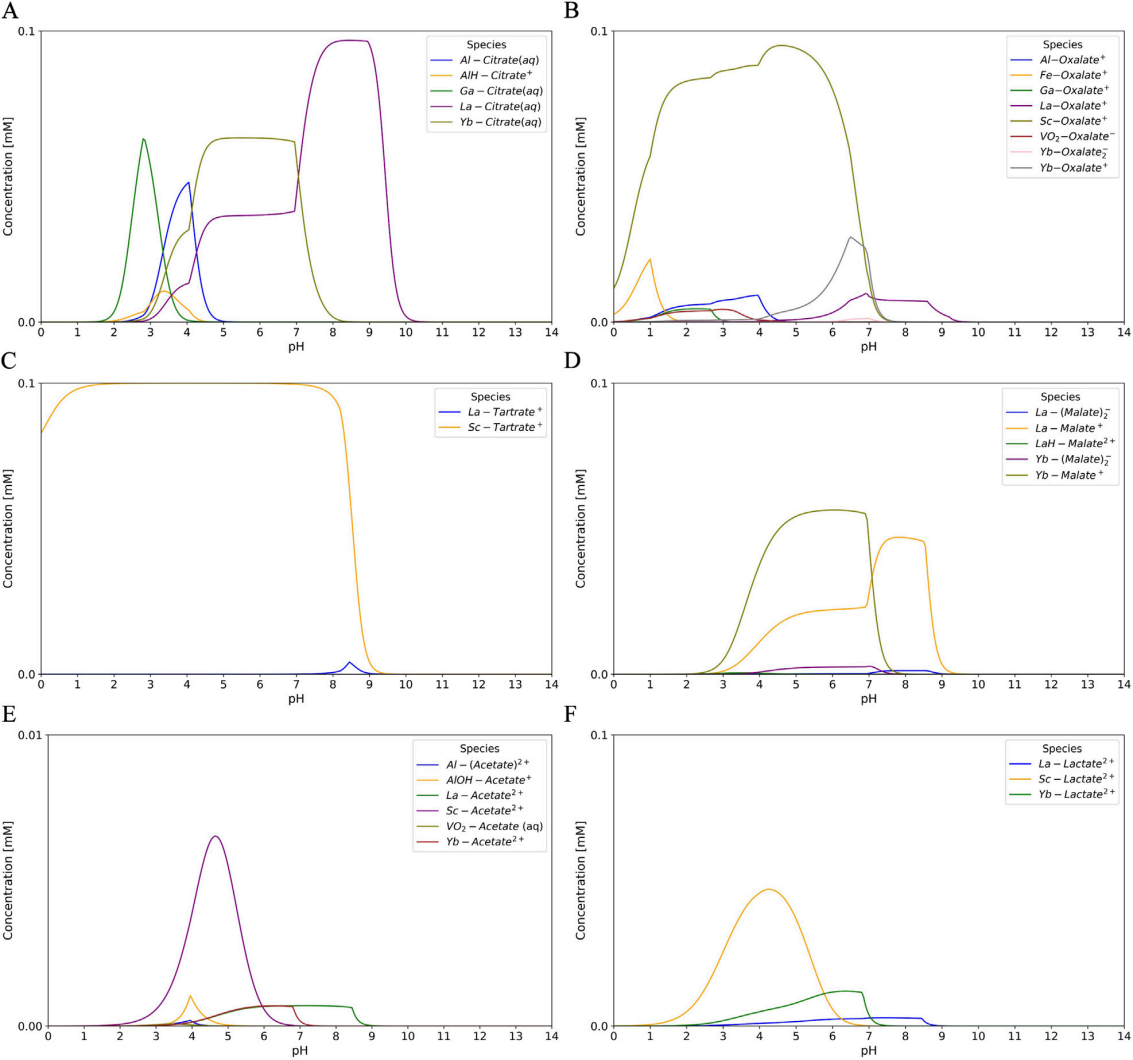
Speciation curves in the liquid phase for organic acid-metal complexes as a function of pH at 25 °C and 1 atm for: **(A)** citric acid, **(B)** oxalic acid, **(C)** tartaric acid, **(D)** malic acid, **(E)** acetic acid, and **(F)** lactic acid. La and Yb are used as representative examples of LREEs and HREEs, respectively. The solubility of organic acid-REEs complexes varies among the individual rare earth elements. Species and concentrations were simulated for each organic acid (0.1 mM) in the presence of Al (0.1 mM), Fe (0.1 mM), La (0.1 mM), Ga (0.1 mM), Yb (0.1 mM), Sc (0.1 mM), Ti (0.1 mM), and V (0.1 mM) in 0.1 M NaCl, using speciation data obtained from Visual MINTEQ (Findlow et al., 1990; Pozdnyakov et al., 2013; Visual MINTEQ, 2021).

Citric acid contains three carboxylic functional groups and is a widely used organic acid in industry. Its commercial production is primarily limited to fungal species such as *Aspergillus niger* and certain yeast species. Citric acid has three acidic protons, which enables it to form complexes with metals of different valence states present in or derived from the bauxite residue, including Al, Fe, Ti, REEs, Ga, and V (Matzapetakis et al., 2001; Tsaramyrsi et al., 2001; Vukosav et al., 2012; Janusz et al., 2020; Meng et al., 2023). The species present depend on the environmental pH, with LREE-citrate complexes predominant at alkaline conditions whilst the concentration of Al-citrate species increases in acidic conditions (Figure 5A). The solubility of citrate-REE complexes depends on the metal-to-ligand ratio, and the specific characteristics of each REE, with HREEs exhibiting greater dissolution than LREEs due to the formation of more stable complexes, thereby favoring the selective extraction of HREEs (Meng et al., 2023; Wen et al., 2024).

Oxalic acid plays a crucial role in the organisms’ tolerance to metals, nutrient acquisition, mineral weathering, Ca regulation, homeostasis, and defense mechanisms (Amenaghawon et al., 2024; Grąz, 2024). While bacteria can produce oxalic acid, fungi are preferred due to their higher productivity (Hamel et al., 1999). Fungal species from the genera *Aspergillus* and *Penicillium*, including *Aspergillus flavus*, *A. niger*, and *Penicillium oxalicum*, are the most commonly used for microbial production of oxalic acid (Amenaghawon et al., 2024). This diprotic acid is used across a wide range of industries, including the mining sector for metal recovery (Xia and Griffith, 2018). Metal oxalates tend to be sparingly soluble; however, Al, Sc, and Fe oxalate complexes are observed in the liquid phase under strongly acidic conditions (Figure 5B). This makes oxalic acid an effective precipitating agent for recovering critical metals from the pregnant leach solution following the bioleaching of bauxite residue (Gadd, 1999; Nawab et al., 2022). The differential solubility of LREE- and HREE-oxalate complexes can be exploited to optimize the selective separation of HREEs, with preferential dissolution and mobilization into the aqueous phase occurring during the redissolution step after oxalate precipitation (Prodius et al., 2020).

Tartaric acid is industrially produced through three principal approaches: extraction of winemaking by-products using dilute hydrochloric acid or hot water; chemical catalysis; and hybrid chemical-enzyme catalysis utilizing petroleum-derived compounds and maleic acid, respectively (Li X. et al., 2024; Li et al., 2025). Despite its natural abundance and industrial importance, large-scale biosynthesis of this organic acid remains challenging. The development of a fully biological route for tartaric acid production is constrained by incomplete pathways in native microorganisms and by insufficient understanding of fungal biosynthetic mechanisms. Depending on the pH, tartaric acid can exist in three different species. It has been reported that under strongly acidic conditions, soluble complexes are formed with Al, and its concentration decreases as the pH increases, allowing the formation of soluble complexes with HREEs and LREEs, with LREEs reportedly exhibiting greater dissolution than HREEs in acidic conditions (pH = 2.6) (Desroches et al., 2000; Nayl et al., 2020; Lallemand et al., 2022). During the simulation, no Al- or Fe-tartrate complexes were observed; instead, mainly Sc-tartrate complexes were detected, owing to the higher stability of the complexes they form (Smith et al., 2004) (Figure 5C). This characteristic renders tartaric acid an effective agent for the efficient recovery of REEs from bauxite residue, while minimizing interference from contaminants.

Although considerable progress has been made in the biosynthesis of malic acid through fermentation, it has not yet been adopted on an industrial scale. Like tartaric acid, malic acid is a diprotic acid, and forms soluble complexes with metals such as Al, Fe, Ti, and REEs (Venturini-Soriano and Berthon, 2001; Vukosav et al., 2010; Katsumata et al., 2011; Zabiszak et al., 2024). It has been reported that under strongly acidic conditions (pH <3), malic acid forms soluble Al complexes that can contaminate the leachate (Drábek et al., 2015). During the simulation, at higher pH values (2.3–8.8), complexes with HREEs and LREEs become more stable and abundant, with HREEs predominating under acidic to near-neutral conditions, while LREEs are more abundant at neutral to mildly alkaline pH (Smith et al., 2004). This behavior enhances the selective recovery of critical metals from bauxite residue (Figure 5D).

Gluconic acid is a monoprotic acid capable of forming water-soluble complexes with metal ions, exhibiting excellent chelating properties at alkaline pH (Yadav et al., 2022). It is currently produced commercially using filamentous fungal species such as *A*. *niger* and *Penicillium* spp., which oxidize glucose. However, current databases used in speciation models of organic acids do not include gluconic acid, making it difficult to represent its metal speciation.

Acetic acid is a monoprotic acid that is primarily produced by chemical methods, although it can also be generated biologically through acetogenesis (anaerobic bacteria converting CO_2_ and H_2_ to acetate via the Wood-Ljungdahl pathway), acetic fermentation (oxidation of ethanol by acetic acid bacteria), or yeast fermentation (where acetic acid can be a by-product of sugar metabolism) (Merli et al., 2021). While Fe-acetate complexes tend to be insoluble, Al complexes are soluble under acidic conditions. Complexes of REEs, V, and Sc with acetate are generally stable under neutral to mildly alkaline conditions (Figure 5E).

Lactic acid is a monoprotic acid, that can be produced through two main pathways: chemical synthesis or fermentation of various carbon sources by different organisms, primarily lactic acid bacteria. This organic acid can form soluble complexes with critical metals under neutral and slightly alkaline conditions. Al-lactate complexes are water-soluble and primarily form under extremely acidic conditions; however, under the simulation conditions, mainly Sc-lactate complexes were observed (Figure 5F). Lactate tends to form soluble complexes with REEs in the form of mono-, bi-, or tri-lactate complexes at pH above 3, in a selective manner, avoiding complexation with Al and Fe. This makes it a potential bioleaching agent for the selective recovery of critical metals from bauxite residue (Couturier et al., 2025).

### 3.4 Microorganisms implicated in bioleaching of bauxite residue

A range of chemoautotrophic and chemoheterotrophic microorganisms has been studied for their potential to bioleach critical metals from bauxite residue. Because of the highly alkaline nature of bauxite residue and the absence of suitable energy sources, chemoautotrophs commonly used in sulfide ore bioleaching through reduction-oxidation reactions are largely ineffective under bauxite residue leaching conditions due to their acidophilic nature (Burgstaller and Schinner, 1993). In contrast, chemoheterotrophs are better suited, as they can thrive in nutrient-supplemented media and produce key metabolites such as organic acids, amino acids, and proteins that promote metal solubilization via acidolysis and complexolysis. These compounds can form complexes with toxic elements present in the bauxite residue, thereby reducing metabolic stress and promoting microbial survival and activity (Valix and Loon, 2003; Santini et al., 2015).

Microorganisms selected for the bioleaching of bauxite residue face considerable challenges; for instance, they must tolerate high metal concentrations and maintain an internal pH lower than that of their environment. This requires physiological adaptations, such as enhanced proton uptake mechanisms and cell membrane modifications to resist osmotic stress and continue producing useful metabolites (Naykodi et al., 2022; Sarma et al., 2023). Despite these challenges, several microbial species have been identified as candidates for the effective leaching of bauxite residue, including acetic acid bacteria (*Acetobacter tropicalis*, *Gluconobacter oxydans*), lactic acid bacteria (*Lactobacillus pentosus*), and filamentous fungi such as *A*. *niger*, and *P. oxalicum*. The metabolic pathways and leaching efficiencies of these organisms will be discussed in the following sections.

### 3.5 Metabolic pathways for organic acid synthesis

**FIGURE 6:**
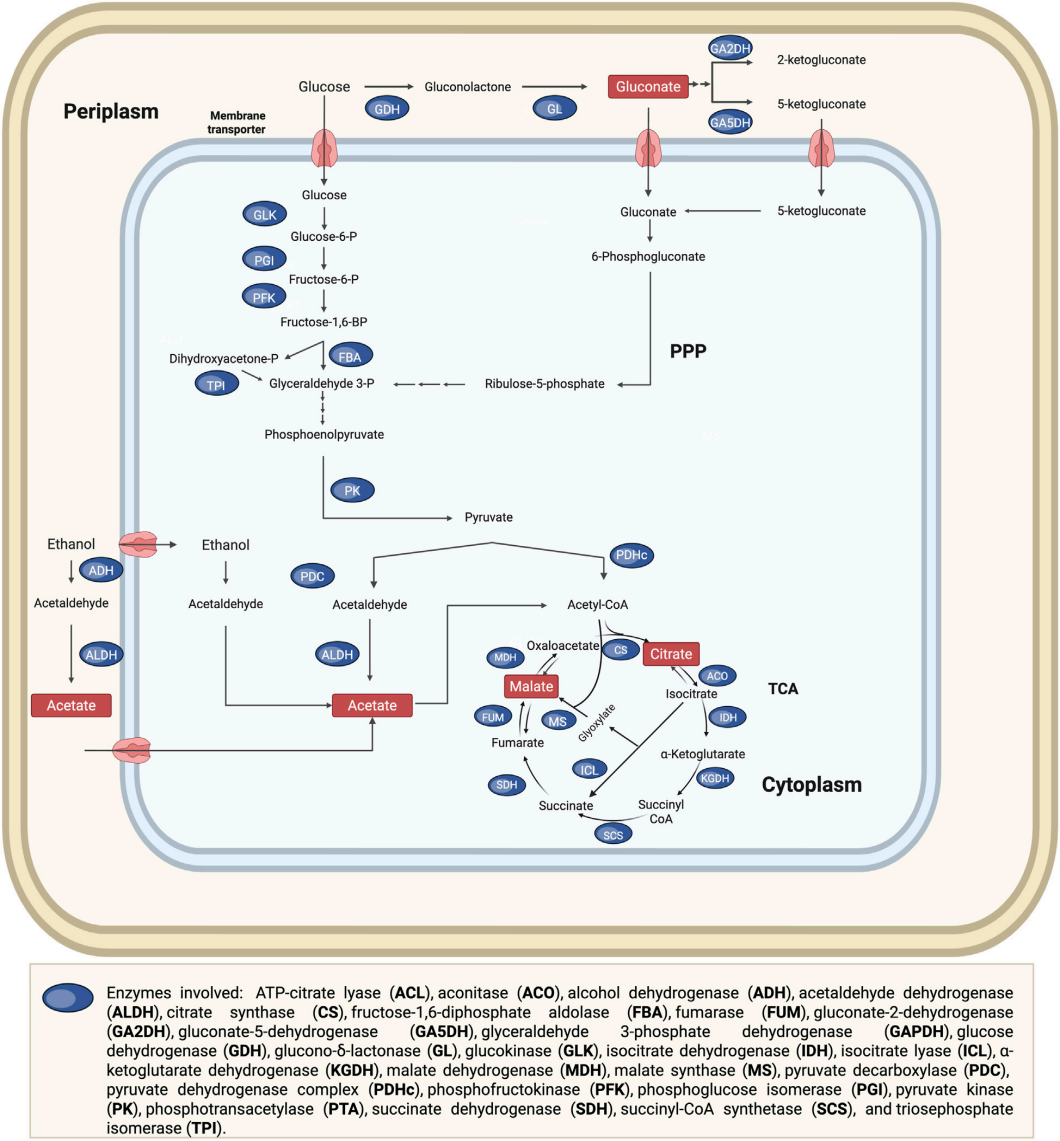
Metabolic pathways for organic acid production in acetic acid bacteria, showing the tricarboxylic acid (TCA) cycle and pentose phosphate pathway (PPP). Multiple arrows indicate that various metabolic reactions are involved. Adapted from (Mamlouk and Gullo, 2013; Matsushita et al., 2016; De Vuyst et al., 2017).

Various acetic acid bacteria (AAB) and lactic acid bacteria (LAB) have been employed for the extraction of critical metals from bauxite residue. In acetic acid bacteria, the production of acids involves the Embden-Meyerhof-Parnas pathway (EMP pathway, also known as glycolysis), the tricarboxylic acid (TCA) cycle, pentose phosphate pathway (PPP) and the glyoxylate cycle (GLOX). Acetic acid can be produced via two main routes. When ethanol is used as the substrate, it is first oxidized to acetaldehyde by alcohol dehydrogenase (ADH), and then to acetic acid by acetaldehyde dehydrogenase (ALDH). When glucose is the carbon source, acetic acid is produced via pyruvate decarboxylation to acetaldehyde, followed by oxidation to acetate by ALDH. Organic acids such as citrate, malate, and fumarate are produced as intermediates of the TCA cycle (Figure 6) (Mamlouk and Gullo, 2013; De Vuyst et al., 2017). However, not all acetic acid bacteria possess a fully functional TCA cycle. For example, *Gluconobacter* species lack key enzymes, α-ketoglutarate dehydrogenase and succinate dehydrogenase, resulting in an incomplete TCA cycle. Therefore, they rely on periplasmic membrane-bound dehydrogenases for oxidative reactions, the PPP for the catabolism of sugars and their derivatives, and a partial TCA cycle for the biosynthesis of metabolic intermediates (Hanke et al., 2013; Bringer and Bott, 2016).

**FIGURE 7:**
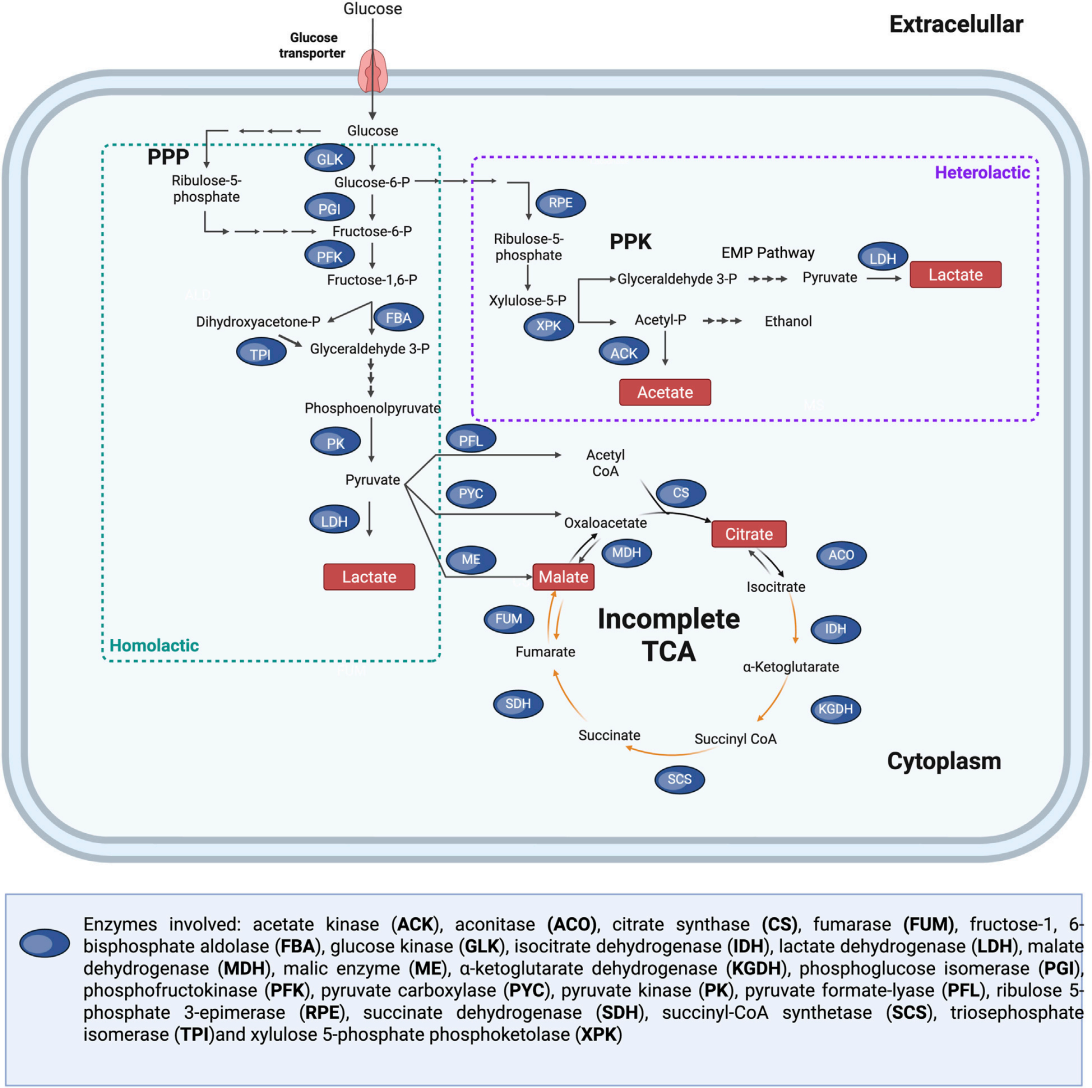
Metabolic pathways involved in organic acid production in lactic acid bacteria (LAB), highlighting the incomplete tricarboxylic acid (TCA) cycle, pentose phosphate pathway (PPP), and pentose phosphoketolase (PPK) pathway. Orange arrows indicate enzymes commonly absent in LAB, while multiple arrows denote the involvement of multiple metabolic reactions. Adapted from (Zotta et al., 2017; Abedi et al., 2020).

Lactic acid bacteria rely on fermentation pathways for energy, and their production of organic acids depends on whether the organism follows a homofermentative or heterofermentative metabolic pathway. Hexoses, such as glucose, are typically metabolized via the homofermentative pathway primarily through the EMP pathway, although some species can also utilize the PPP for lactate production. In contrast, glucose can be converted into lactate along with acetate and/or ethanol through the heterofermentative pathway, which involves the pentose phosphoketolase (PPK) pathway. In this pathway, glyceraldehyde-3-phosphate is subsequently metabolized to pyruvate via the EMP pathway (Zotta et al., 2017; Abedi et al., 2020) (Figure 7). Species from *Lactobacillus* and *Bacillus* are examples of heterofermentative lactic acid-producing bacteria.

LAB lack a complete TCA cycle due to the absence of isocitrate dehydrogenase, α-ketoglutarate dehydrogenase, succinyl-CoA synthetase, and succinate dehydrogenase. However, some LAB species can convert pyruvate into oxaloacetate or malate, allowing entry into the incomplete TCA cycle and enabling the production of fumaric acid and citric acid at low extracellular levels (Morishita and Yajima, 1995).

**FIGURE 8:**
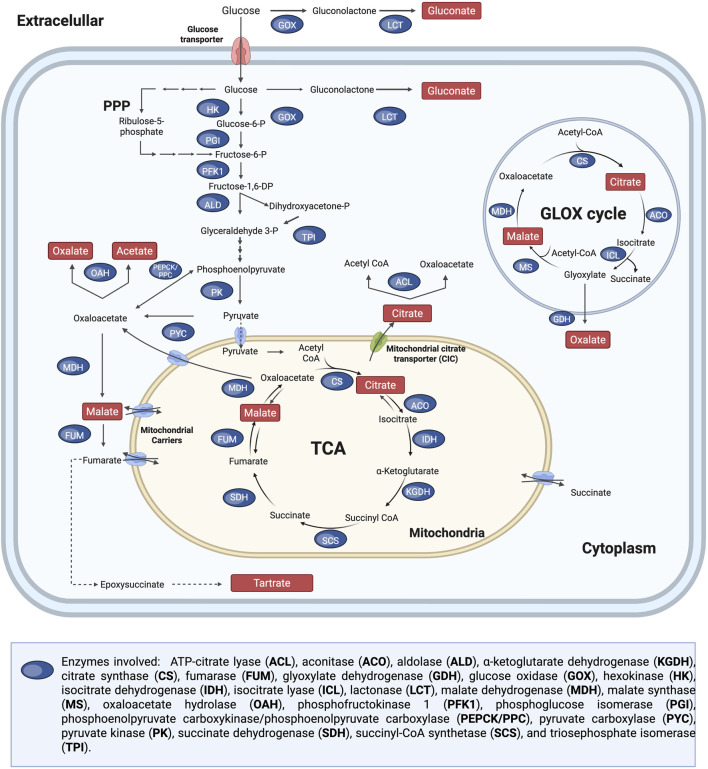
Metabolic pathways for organic acid production in filamentous fungal species, showing the tricarboxylic acid (TCA) cycle, pentose phosphate pathway (PPP), and the glyoxylate cycle (GLOX). Multiple arrows indicate that various metabolic reactions are involved. Dotted lines indicate hypothetical metabolic pathways that have not yet been experimentally validated. Adapted from (Izcapa-Treviño et al., 2009; Kobayashi et al., 2014; Hronská et al., 2017; Ma et al., 2022; Yadav et al., 2022; Khandelwal et al., 2023; Wu et al., 2023; Książek, 2024; Li X. et al., 2024).

Organic acid production in filamentous fungi involves multiple metabolic pathways and cellular compartments. These include glycolysis (cytosol), the TCA cycle (mitochondria), the pentose phosphate pathway (cytosol), and GLOX, which occurs in the peroxisomes (Figure 8). Citric acid production primarily involves the TCA cycle, GLOX cycle, and PPP. Oxalic acid is synthesised via three mechanisms: oxidation of glyoxylic acid by glyoxylate dehydrogenase (GDH), pyruvate carboxylation in the cytoplasm and the hydrolysis of intermediates from the TCA cycle. In *A. niger*, only the TCA and pyruvate pathways are active, as no GDH activity has been observed. Tartaric acid production has previously been demonstrated in filamentous fungal strains (Izcapa-Treviño et al., 2009; Li et al., 2016), however, to the best of the authors’ knowledge, the corresponding metabolic pathway has not yet been experimentally validated. Malic acid biosynthesis in fungi is produced via three pathways: cytoplasmic pyruvate carboxylation, TCA cycle intermediates, and the GLOX cycle. Gluconic acid is produced via glucose oxidation by glucose oxidase (GOX), primarily found in the cell wall and extracellular fluid of *Aspergillus* and *Penicillium*. Gluconolactone hydrolysis occurs spontaneously at neutral/alkaline pH or via lactonase (LCT) in acidic conditions.

### 3.6 Bioleaching of critical metals

Organic acids, whether chemically synthesized or produced biologically through one-step or two-step cultivation of acid-producing microorganisms, have been employed for the extraction of critical metals from bauxite residue (Borra et al., 2015; Gu et al., 2020). Notably, the use of biotically produced organic acids is a promising approach, as these acids have been reported to exhibit superior leaching capacity for critical minerals compared to chemically pure acids, even at lower molarities (Aung and Ting, 2005; Amiri et al., 2011; Van Wyk et al., 2024). Moreover, the biotic production of organic acids offers substantial advantages at an industrial scale, including lower production costs compared to purchasing commercial acids. In addition, several microorganisms capable of generating high concentrations of organic acids can utilize industrial waste as a nutrient source, thereby enhancing the circularity of the process and further reducing operational costs (Sawant et al., 2018; Egbe et al., 2022; Mahgoub et al., 2022).

The extraction of critical minerals from bauxite residue using naturally enriched autochthonous fungi and bacteria, without external microbial inoculation, was demonstrated in a sequencing batch reactor (Cozzolino et al., 2024). This process resulted in recoveries of 65.2% for Nd, 19.5% for Ce, 30.2% for Y, 34.4% for Sc, and 21.4% for La, with minimal Fe leaching (<0.1%) when processed at a 1.5% solid-to-liquid (S/L) ratio. A high concentration of acetic acid, along with an enrichment of acidophilic and neutrophilic bacterial populations, was detected during the fermentation process. These findings support the application of indigenous fungal-bacterial consortia as a promising alternative, highlighting the relevance species such as *P. oxalicum*, previously isolated from bauxite residue and known for their high organic acid production (Liao et al., 2018).

Notably, fungal species employ diverse strategies to survive in extreme environments such as bauxite residue, including the secretion of organic acids that both chelate metal ions and lower waste alkalinity, thereby mitigating toxic effects and facilitating metal recovery (Ghorbani et al., 2008; Liao et al., 2018; Qu et al., 2019a; Feigl et al., 2024). As shown in Table 2, the bioleaching capabilities of *A. niger* have been evaluated using one-step, two-step, and spent medium approaches at various bauxite residue concentrations. In the one-step configuration with a 2% w/v bauxite residue concentration, a higher Ti recovery was observed compared to the two-step method, likely resulting from enhanced fungal growth, as indicated by increased biomass, and elevated excretion of leaching agents (mainly organic acids) driven by metal-induced stress. In contrast, the spent medium leaching configuration resulted in the lowest metal recovery among all tested approaches (Vakilchap et al., 2016).

**TABLE 2:** Fungal and bacterial recovery of critical minerals from bauxite residue via acidolysis and complexolysis mechanisms. Bauxite residue (BR) concentrations are indicated as percentage w/v, unless otherwise specified.

Organism	Steps	BR	Conditions	Efficiency (%)	References
*A. niger*	One-step	2	30 °C, 130 rpm, 30 days	Ti (60), Al (70), Fe (25)	Vakilchap et al. (2016)
*A. niger*	Two-step	2	30 °C, 130 rpm, 30 days	Ti (22), Al (54), Fe (10)	Vakilchap et al. (2016)
*A. niger*	Spent medium	2	30 °C, 130 rpm, 30 days	Ti (11), Al (46), Fe (4)	Vakilchap et al. (2016)
*A. niger* ^a^	Two-step	3	30 °C, 150 rpm, 20 days	Ti (67), Sc (<20), V (88), Al (92)	Pedram et al. (2020)
*A. niger* ^b^	Two-step	3	30 °C, 150 rpm, 20 days	Ti (<60), Sc (∼30), V (31), Al (53)	Pedram et al. (2020)
*A. niger* ^a^	One-step	3	30 °C, 150 rpm, 20 days	V (91), Al (97)	Azimi et al. (2024)
*A. niger*	One-step	1	30 °C, 100 rpm, 20 days	Sc (46)	Kiskira et al. (2023)
*A. niger*	One-step	10	30 °C, 250 rpm, 15 days	Ga (31), Ge (33), V (19), Sc (30), La (16), Eu (23), Yb (44)	Qu et al. (2015)
*P. chrysogenum*	One-step	3	30 °C, 120 rpm	Y (79), La (28), Ce (28)	Ilyas et al. (2021)
*P. tricolor*	Two-step	8.3	30 °C, 120 rpm, 40 days	V (∼34)	Qu et al. (2019a)
*P. tricolor*	Two-step	4	30 °C, 150 rpm, 30 days	Ti (64)	Qu et al. (2022)
*P. tricolor*	One-step	2	30 °C, 120 rpm, 30 days	Y (>70)	Qu and Lian (2013)
*G*. *oxydans* ^c^	Two-step	10	37 °C, 120 rpm, 20 days	Sc (94), La (40), Ce (40), Nd (40), Y (81)	Abhilash et al. (2021)
*G*. *oxydans*	Two-step	0.5	30 °C, 120 rpm, 3 days	Sc (13), Y (41), La (15), Ce (24), Nd (11), Ti (59), Ca (80), Al (68)	Van Wyk et al. (2024)
*Bacillus nitratireducens*	Two-step	0.15	25 °C, 180 rpm, 3 days	Lu (92), Tb (81), Gd (67)	Rus et al. (2024)
*Acetobacter* sp.	One-step	2	30 °C, 120 rpm	Sc (42)	Qu et al. (2019b)
*A. tropicalis*	One-step	1^d^	30 °C, 120 rpm, 20 days	Ti (3), Sc (4), Al (28)	Kiskira et al. (2021)
*L*. *pentosus*	Two-step	20	30 °C, 180 rpm, 8 days	Pr (35), Ce (6), La (1)	Han et al. (2024)
*B*. *foraminis*	One-step	1	40 °C, 160 rpm, 8 days	Y (>70)	Ilkhani et al. (2024)

^a^ Grape-skin extracted *A*. *niger* strain.

^b^ Pistachio shell extracted *A*. *niger* strain.

^c^ Bauxite residue tolerant strain (20% w/v) by adaptive laboratory evolution.

^d^ S/L ratio.

Pedram et al. (2020) reported that the recovery efficiency of individual metals varied depending on the *A. niger* strain and the bioleaching configuration used. For V extraction, the one-step process proved to be the most effective, regardless of the strain used. In contrast, Ti recovery was enhanced in both fungal strains under the two-step bioleaching approach. Overall, the results indicated that adding bauxite residue at the time of spore inoculation led to higher metal extraction compared to its addition after 3 days of incubation, likely due to better fungal adaptation during the initial growth phase.

Although fungal species are generally preferred for industrial-scale applications, due to their typically higher organic acid yields, ability to grow on low-cost substrates, effectiveness across a wide pH range (2–9), and greater resilience in alkaline environments, the use of bacterial species for the bioleaching and neutralization of bauxite residue has also been widely reported (Dezam et al., 2017; Dusengemungu et al., 2021; Gnanasekaran et al., 2022; Mahgoub et al., 2022). As shown in Table 2, the bioleaching potential of *L. pentosus* was evaluated using pre-treated rice straw as a carbon source, achieving recovery efficiencies of 2.9% for Ti and 3.8% for Sc, although 28% of the contaminant Al was leached (Han et al., 2024).

The performance of *L. pentosus* under liquid batch fermentation at high bauxite residue concentrations (20% w/v), using glucose as the carbon source, was compared to two previously reported highly efficient bioleaching species: *A. niger* and *G. oxydans*. Under these conditions, neither *A. niger* nor *G. oxydans* exhibited substantial growth or organic acid production, resulting in negligible leaching efficiencies (Han et al., 2024). These findings underscore the importance of optimizing strains with demonstrated high organic acid productivity and leaching efficiency to maintain robust performance under elevated bauxite residue concentrations. Achieving tolerance to such high concentrations is essential for processing larger waste volumes and maximizing the simultaneous recovery of critical metals.

Bioprocessing of bauxite residue can be integrated with hydrometallurgical pretreatment methods. For example, bioleaching of pretreated bauxite residue using *Bacillus foraminis* was evaluated after removing 98.4% of Fe and Al, and 80% of Ti. Leaching of the remaining fraction by this bacteria achieved significantly higher recoveries compared to direct leaching of untreated residue: 35% Pr, 6.3% Ce, and 1% La from treated bauxite residue, versus 0.6% Pr, 0.7% Ce, and 0.3% La from untreated residue (Ilkhani et al., 2024).

As shown in Table 2, several microbial species have been tested for the extraction of critical metals, achieving high recovery efficiencies. However, these microorganisms may also leach undesirable elements such as Fe and Al. These findings highlight that bioleaching is not inherently selective, and the performance of each bioleaching agent or microorganism must be evaluated based on the specific composition of the bauxite residue. At the industrial scale, bioleaching faces challenges such as increasing leaching rates, reducing extraction time, and increasing pulp density, which is considered a critical factor for economic viability (Pathak et al., 2017). Therefore, various strategies are employed to optimize bauxite residue bioleaching, aiming to increase metal recovery rates and extents through parameter optimization, and to improve microbial tolerance or enhance leaching agent production through organism optimization.

#### 3.6.1 Parameters optimization

The solubilization efficiency of critical metals during bioleaching is governed by several factors, including the specific bioleaching steps, preculture time, inoculation size, solid-to-liquid ratio, duration of bioleaching and the composition of bauxite residue as well as ease of accessibility of metals (Table 2). Furthermore, the mobilization of critical metals from the solid phase is influenced by environmental parameters, including temperature and pH, as well as by the stability of metal-organic acid complexes (Kiskira et al., 2021). Determining optimal conditions is inherently a time-consuming and complex task, as it is typically conducted by varying a One Factor at a Time (OFAT) and evaluating the effect of individual factors separately, often overlooking interactions between them. To address these challenges and reduce the need for iterative, costly experimentation, computational and statistical modeling approaches, collectively referred to as Design of Experiments (DoE), can be employed to predict optimal parameters while requiring fewer experimental data points. Within this framework, Response Surface Methodology (RSM) is a widely used statistical and mathematical DoE technique, recognized as one of the most effective and standardized methods for the design, modeling, and optimization of multivariable processes, enabling the assessment of interaction effects among variables using data from strategically designed and fewer experiments (Kim et al., 2023). RSM can also be integrated with machine learning and artificial intelligence techniques to enhance optimization efforts and to develop predictive models of bioleaching behavior based on experimental data (Naseri et al., 2023; Saldaña et al., 2023; Trivedi and Hait, 2024).

RSM has been extensively employed to optimize bioleaching processes for low-grade ores, industrial wastes, and electronic wastes. The recovery of copper (Cu) from low-grade sulfide ores was optimized using *Acidithiobacillus ferrooxidans* and *Acidithiobacillus thiooxidans* by adjusting key operational parameters, including pH, S/L ratio, and agitation rate, achieving a Cu recovery of 69.9% (Naderi et al., 2017)*.* Similarly, RSM-based optimization of Cu extraction from sewage sludge using *A. thiooxidans* was conducted by evaluating the effects of, and optimizing, process variables such as initial sulfur concentration, S/L ratio, and initial pH. Under the optimal conditions suggested by the model, a maximum experimental Cu recovery of 85.3% was achieved, which exceeded the maximum predicted value of 80.9% (Rastegar et al., 2024).

Fungal bioleaching using *A. niger* for the simultaneous recovery of Cu and nickel (Ni) from printed circuit boards has been optimized using RSM (Arshadi et al., 2020). The parameters evaluated included the S/L ratio, inoculum size, bioleaching pH, and the number of bioleaching steps. Experimental recoveries reached approximately 97% for Cu and 74% for Ni, while the model predicted recoveries of 100% and 80%, respectively, with the experimental values falling within the 90% confidence intervals of the predictions. This strong agreement underscores the potential of statistical and mathematical approaches for the optimization of bioprocesses.

To date, most studies employing these approaches for bauxite residue have focused on predicting the properties of the alumina refinery residue (Yang F. et al., 2025), evaluating the properties of construction materials derived from it (Promentilla et al., 2016; Pratap, 2024), assessing its environmental remediation capabilities (Martins et al., 2020; Rushendra Revathy et al., 2021) and determining optimal conditions for non-biological metal extraction from the residue (Shoppert et al., 2022; Pilla et al., 2025). However, the optimization of bioleaching conditions for bauxite residue has been largely overlooked. Qu et al. (2019a) used RSM to determine the optimal sucrose concentration, inoculum size, bauxite residue concentration, and pre-culture duration required to maximize biomass accumulation, organic acid production, and V extraction using *A. niger* and *P. tricolor*. The analysis revealed that the optimal conditions varied depending on the specific target. Under the optimized conditions, V bioleaching achieved recoveries of 32.4% and 34% for *A. niger* and P*. tricolor*, respectively. Similarly, Azimi et al. (2024) employed an artificial neural network optimization approach to enhance the recovery of Al and V by adjusting key process variables, including the *A. niger* strain (isolated from pistachio shells or grape skins), bioleaching steps, grinding time of the sample, S/L ratio, and leaching time. The method predicted optimal recoveries of 97.5% for Al and 88.7% for V, which were experimentally validated through bioleaching tests, with actual recoveries of 97.1% and 90.3%, respectively, and showing a relative error of less than 1.8%. This highlights an underexplored opportunity to investigate the potential of machine learning-based techniques for optimizing the recovery of critical metals from alumina refinery residue.

#### 3.6.2 Organism optimization

##### 3.6.2.1 Adaptive laboratory evolution

Bioleaching of bauxite residue has been evaluated in different configurations and at various pulp densities, generally showing better recoveries in direct leaching than in spent-medium use (Qu and Lian, 2013). However, decreased leaching performance is observed when the pulp density reaches 10%, as high solid loads inhibit microbial growth and lead to low leaching efficiencies in both one-step and two-step configurations. This effect is attributed to the high salinity, heavy metals, and alkalinity of the bauxite residue (Qu and Lian, 2013; Qu et al., 2013; Han et al., 2024; Van Wyk et al., 2024). To enhance robustness against bauxite residue toxicity, a new generation of organisms has been developed using phenotype-driven experimental methodologies, such as Adaptive Laboratory Evolution (ALE).

**FIGURE 9:**
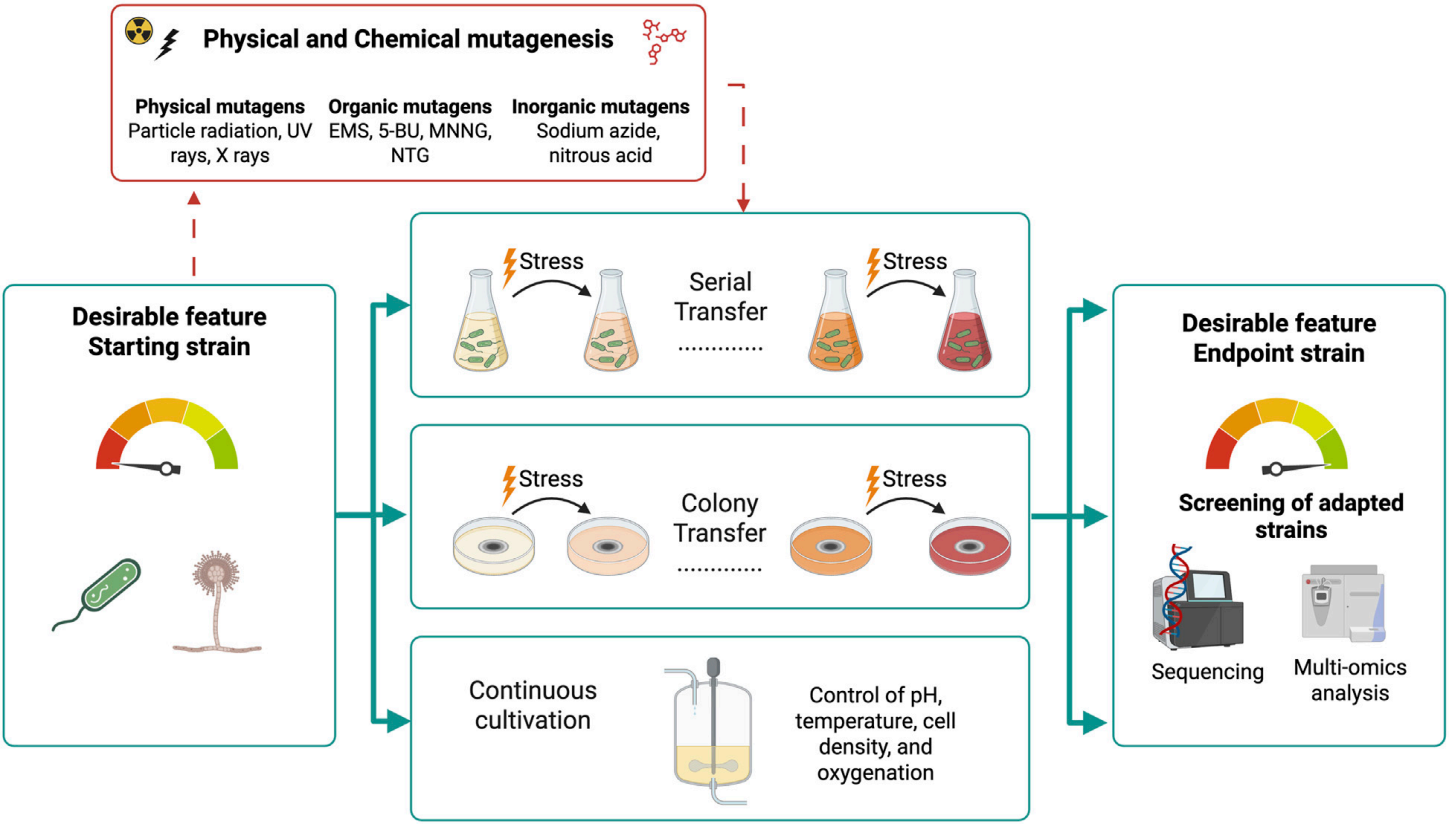
Strain optimization via adaptive laboratory evolution (ALE). The optional use of mutagenic agents to increase genetic diversification in bacterial and fungal species during ALE is indicated by dotted arrows. Commonly used physical, inorganic, and chemical mutagens, such as ethyl methanesulfonate (EMS), 5-bromouracil (5-BU), 1-methyl-3-nitro-1-nitrosoguanidine (MNNG), and N-methyl-N′-nitro-N-nitrosoguanidine (NTG), are shown. Adapted from (Lotfy et al., 2007; Karanam and Medicherla, 2008; Vu et al., 2009; Irfan et al., 2011; Barreiro et al., 2012; Qi et al., 2014; Patyshakuliyeva et al., 2016; Burlacu et al., 2017; Hirasawa and Maeda, 2022; Mavrommati et al., 2022; Oshoma et al., 2022; Zhao et al., 2022; Yang et al., 2024).

ALE is an effective method that harnesses the natural adaptability of microorganisms to specific environmental conditions. Under defined cultivation conditions, cells exhibiting a desired phenotype gain a selective advantage, leading to the development of optimized strains that can subsequently be isolated through targeted screening (Dragosits and Mattanovich, 2013) (Figure 9). Over successive generations, beneficial mutations accumulate across genes and regulatory regions, enhancing overall cellular functions and resulting in optimized strains (Portnoy et al., 2011). ALE also reveals the genetic and metabolic pathways underlying microbial adaptation, as evolved strains are analyzed using genome sequencing, transcriptomics, proteomics, and metabolomics, thereby guiding the development of more robust bioleaching strains. Moreover, ALE is often preferred because it faces fewer regulatory restrictions than genetically engineered strains.

ALE can be conducted via serial transfer, colony transfer, or continuous cultivation, chosen based on the microorganism’s growth characteristics and adaptation goals (Figure 9). Serial transfer (or serial batch cultivation through sub-culturing) is widely used for bacteria due to its ease of automation and scalability, allowing selection of cells that tolerate progressively higher stress levels. Colony transfer is more suitable for fungi, which tend to grow as pellets or biofilms, where spores are harvested from solid media and exposed to selective pressures. Continuous cultivation in bioreactors offers precise control over environmental parameters (e.g., pH, oxygen, nutrient supply, cell density) but is less common for filamentous species due to reproducibility challenges and likelihood of biomass retention (Hirasawa and Maeda, 2022; Mavrommati et al., 2022).

In metal recovery contexts, ALE has been successfully applied to acidophilic archaea and bacteria, improving acid tolerance and bioleaching efficiency. For instance, *Metallosphaera sedula* and *Acidithiobacillus* spp. strains adapted via ALE exhibited increases of 23.8% and 35.1%, respectively, in bioleaching efficiency relative to the parental strains (Feng et al., 2015; Ai et al., 2016). Acid-tolerant consortia comprising *Leptospirillum ferriphilum*, *Sulfobacillus thermosulfidooxidans*, and *Ferroplasma thermophilum* have also been adapted for acid resistance and enhanced Fe oxidation, resulting in an increase in iron extraction from 26% to 55%, depending on pH (Liu et al., 2019). Directed evolution of *L*. *ferriphilum* for adaptation to extreme acidity enabled survival at pH 0.7 and showed a 2-fold increase in leaching efficiency at pH 1.0 (79.5%) compared to the wild type (39.5%) (Liu and Zhou, 2022). Similarly, directed evolution of *L. ferriphilum* to withstand inhibitory substances such as thiocyanate has been demonstrated while maintaining or improving ferrous iron oxidation efficiency and associated pyrite leaching in the gold biooxidation circuit (Edward et al., 2018). Directed evolution of *L. ferriphilum*, *Acidithiobacillus caldus*, and *Acidiplasma cupricumulans* to tolerate Cu for leaching of elemental forms of base metals from e-waste has also been demonstrated (Maluleke et al., 2024).

For fungal bioleaching, *A. niger* adapted to increasing concentrations of lithium-ion battery powder (up to 1% w/v) produced higher levels and altered profiles of organic acids, enhancing metal recovery of Al, Ni, Cu, manganese and cobalt by 4%–83% compared to the non-adapted strain (Bahaloo-Horeh et al., 2018). Similarly, *A. niger* and *Penicillium simplicissimum* adapted to 5% w/v bauxite residue through serial subculturing achieved Al recovery of 97%–98% (Shah et al., 2020). An adapted *G. oxydans* strain exposed to 20% w/v bauxite residue showed Sc leaching efficiencies between 70% and 80%, surpassing the 58%–70% range of the non-adapted strain (Abhilash et al., 2021).

Despite showing promising results, ALE remains underutilized for developing bacterial and fungal strains with enhanced tolerance to bauxite residue, as the concentrations achieved thus far are insufficient to ensure economic feasibility of the process. Moreover, its potential to improve bioleaching efficiency for critical metals in this material is not yet fully explored. Existing research suggests that higher pulp densities of bauxite residue reduce bioleaching efficiency for Ti, Ga, Va, and REEs in non-adapted strains compared to adapted ones (Abhilash et al., 2021), highlighting ALE as a promising strategy to overcome this limitation by generating more robust and efficient strains, as demonstrated for acidophilic metal leaching (Maluleke et al., 2024).

##### 3.6.2.2 Genetic engineering techniques

In contrast to ALE, genetic engineering tools provide rational design through genetic modifications, enabling the optimization of resource usage, reduction of metabolic impact, and enhancement of productivity. Tools such as gene knock-out and knock-in, heterologous gene expression, and the overexpression of target genes via recombinant DNA technology enable the targeted enhancement of pathways involved in stress responses, the microbe-metal interaction, and the biosynthesis of bioleaching agents. For example, transcriptomic analysis provides insights into changes in gene expression and metabolic activity under stress conditions, enabling the identification of genomic targets that could be engineered to improve stress tolerance to bauxite residue (Huang et al., 2022; Jahan et al., 2024).

**FIGURE 10:**
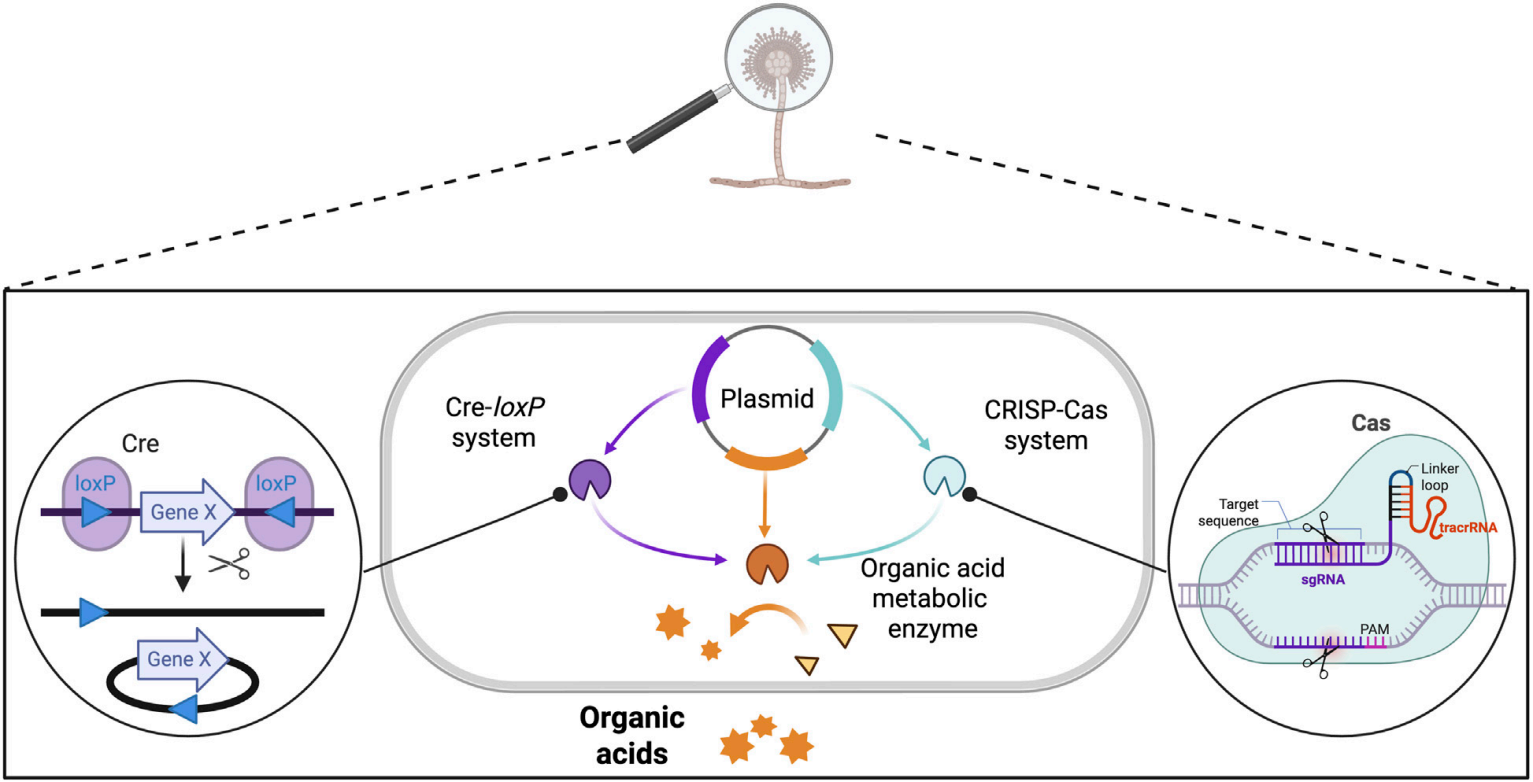
Genetic engineering strategies for enhancing organic acid production in bacteria and fungi. CRISPR-Cas uses RNA-guided Cas nucleases to induce precise DNA double-strand breaks, which are repaired by cellular DNA repair pathways. The Cre-*loxP* system employs Cre recombinase and *loxP* sites, requiring prior genomic integration to enable targeted gene insertion, deletion, or modulation of expression. Adapted from (Mizutani et al., 2012; Zhang et al., 2013; Nishikawa et al., 2016; Wang et al., 2019; Shen et al., 2024).

Different techniques can be utilized for inducing genetic modifications in bioleaching organisms. Plasmid-based methods remain the standard technique for delivering DNA into target organisms, relying on the incorporation of circular DNA sequences to induce production of target proteins (Nishikawa et al., 2016). These systems enable both endogenous and foreign gene expression and can be used to deliver genome editing components such as CRISPR-Cas or tools for site-specific recombination and conditional gene modification (e.g., Cre-*loxP*) (Figure 10). For readers interested in detailed background on genetic modification techniques in bioleaching bacteria, we refer to comprehensive reviews by Chen et al. (2022) and Jung et al. (2022), which cover the fundamentals and recent advances in this field.

CRISPR interference (CRISPRi), which uses catalytically inactive Cas proteins (dCas9 or dCas12a), allows reversible repression of target genes without permanent DNA modifications. For instance, CRISPRi has been applied in *A*. *ferrooxidans* to suppress key sulfur oxidation genes, demonstrating control over metabolic pathways relevant to bioleaching efficiency (Jung et al., 2024). The Cas12a-based systems provide advantages including multiplex editing capability and lower toxicity, suitable for acidophilic bacteria with high GC content genomes. In addition, CRISPR-mediated genome editing in *G*. *oxydans* recently yielded strains with improved REEs bioleaching performance through targeted genetic modifications (Schmitz et al., 2025). These developments highlight the growing potential of CRISPR technologies to engineer bioleaching bacteria for enhanced metal recovery and process optimization.

Studies aiming to enhance organic acid production by genetic engineering techniques are shown in Table 3 (Mizutani et al., 2012; Zhang et al., 2013). Expression plasmids have been extensively used to metabolically engineer AAB, LAB, and *A*. *niger* to overexpress genes involved in organic acid synthesis. For example, overexpression of PQQ-ADH subunits I and II in *Acetobacter pasteurianus* increased acetic acid production from 52.2 g/L to 61.4 g/L (Wu et al., 2017). Expression of glycolytic pathway genes in *Lactobacillus brevis* improved lactic acid yield and glucose conversion efficiency from 0.74 to 1.16 mol/mol (Guo et al., 2014). In *A. niger*, one of the most important industrial organisms for organic acid production, the overexpression of genes encoding alternative oxidase and oxaloacetate hydrogenase boosted oxalic acid production from 21 g/L to 28 g/L and shortened the production time. Recent efforts using Cre-*loxP* and CRISPR-Cas9 systems have also shown promising results for further enhancing organic acid yields. Despite this, the use of genetic engineering tools specifically for enhancing the production of bioleaching agents and evaluating their effectiveness in extracting metals from bauxite residue has not yet been explored.

**TABLE 3:** Examples of genetic modification approaches applied to enhance organic acid production in bacterial and fungal species.

Organism	Acid	System	Encoding Gene(s)	Optimization	References
*A. pasteurianus*	Acetic	Expression plasmid	*adhA, adhB*	Production increased from 52.2 g/L to 61.4 g/L	Wu et al. (2017)
*Acetobacter aceti*	Acetic	Expression plasmid	*aatA*	Production increased from 103.7 g/L to 111.7 g/L	Nakano et al. (2006)
*G. oxydans*	Gluconic	Expression plasmid	*gdh*	11% increase in production	Merfort et al. (2006)
*L. brevis*	Lactic	Expression plasmid	*pfkA, fbaA*	Yield increased from 0.74 mol/mol to 1.16 mol/mol ^a^	Guo et al. (2014)
*A. niger*	Oxalic	Cre-*loxP* system	*oahA*	3.1-fold increase in production	Xu et al. (2019)
*A. niger*	Oxalic	Expression plasmid	*AoxA, oahA*	Production increased from 21 g/L to 28 g/L	Yoshioka et al. (2020)
*A. niger*	Malic	Cre-*loxP* system	*mstC*, *hxkA*, *pfkA*, *pkiA*	23.6% increase in production	Xu et al. (2020)
*A. niger*	Malic	CRISPR-Cas9 system	Promoter replacement: *fumA* → *PmfsA*	9.0% increase in production	Zhang et al. (2024)
*A. niger*	Malic	Expression plasmid	*dct1*	22.8% increase in production	Cao et al. (2020)
*A. niger*	Citric	Expression plasmid	*hgt1*	7.3% increase in production	Xue et al. (2021)
*A. niger*	Citric	Expression plasmid	*cexA*	5-fold increase in production	Steiger et al. (2019)
*A. niger*	Citric	Cre-*loxP* system	*dct1* ^b^	36.4% increase in production	Cao et al. (2020)

^a^ Yield increased to 1.16 ± 0.03 mol of lactic acid per mol of glucose.

^b^ DCT1 knockout.

Genes/Enzymes: *adhA,* subunit I of PQQ-ADH; *adhB*, subunit II of PQQ-ADH; *aatA*, putative ATP-binding cassette (ABC) transporter; *gdh*, glucose dehydrogenase; *pfkA*, fructose-6-phosphate kinase; *fbaA*, fructose-1,6-biphosphate aldolase; *oahA*, oxaloacetate acetylhydrolase; *aoxA*, alternative oxidase; *mstC*, glucose transporter; *hxkA*, hexokinase; *pfkA*, 6-phosphofructo-2-kinase; *pkiA*, pyruvate kinase; *fumA*, fumarase; *PmfsA*, CaCO_3_-induced promoter; *dct1*, C4-dicarboxylate transport protein (DCT1); *hgt1*, high-affinity glucose transporter (HGT1); *cexA*, citrate exporter (CexA).

###### 3.6.2.2.1 Legal framework of genetic engineering techniques

The legal framework behind genetic engineering techniques must be considered before evaluating their applicability and the requirements for implementation at an industrial scale. These frameworks must also be assessed according to the specific regulations of the country where the techniques will be applied. Gene editing techniques are internationally classified into three categories: site-directed nucleases-1 (SDN-1), site-directed nucleases-2 (SDN-2), and site-directed nucleases-3 (SDN-3), depending on the mechanism used for knockouts, insertions, or modifications of the genome (Box 1) (Thygesen, 2019; Shen et al., 2024; Thygesen, 2024).

BOX 1 **Gene editing categories.**

- Site-Directed Nucleases-1 (SDN-1): the position of the double-strand breaks (DSBs) in the genome is selected but its repair does not utilize a DNA template. DNA substitutions, insertions, and the deletions can be produced with this gene editing technique. The repair mechanism is non-homologous end joining (NHEJ).
- Site-Directed Nucleases-2 (SDN-2): a template is used to repair the DSBs by homology-directed repair (HDR). The template contains a small number of nucleotides different from the DNA sequence.
- Site-Directed Nucleases-3 (SDN-3): a DNA template is used for the repair by HDR or NHEJ. A large DNA sequence is inserted in a targeted genomic location.

According to Australia’s regulatory framework, the Gene Technology Act 2000 (Australian Government, 2024b) and the Gene Technology Regulations 2001 (Australian Government, 2024a), a genetically modified organism (GMO) is any organism, whether plant, animal, or microorganism, whose genetic material has been altered through genetic engineering techniques (Australian Government Department of Health, 2024). However, only certain gene technologies result in the development of GMOs. Under Australian federal legislation, organisms created using SDN-1 are not regulated as GMOs, as they are indistinguishable from those with naturally occurring genetic variations and present the same level of risk. However, organisms created using SDN-2 and SDN-3 are classified as GMOs. To utilize GMOs these organisms, a license must be obtained from the regulatory authority, and strict containment, monitoring, and control measures must be implemented to prevent any unintended release into the environment (Australian Government, 2024b).

In the United States, regulation of genetically modified organisms (GMOs) is shared among the Food and Drug Administration (FDA), the U.S. Department of Agriculture (USDA), and the Environmental Protection Agency (EPA) (Food and Drug Administration FDA U.S, 2024). For applications such as bioleaching, the EPA regulates GMOs under the Toxic Substances Control Act (TSCA). The EPA classifies GMOs based on their risk level, intended function, and the method used to create them. However, organisms developed using SDN-1 and SDN-2 techniques are typically considered non-GMOs. The risks and potential societal benefits of each genetically engineered microorganism must be individually assessed prior to industrial or commercial application under TSCA regulation (Wozniak et al., 2012).

In contrast to the U.S., the European Court of Justice considers all organisms obtained through new genomic techniques, including all types of site-directed nucleases, as GMOs under European Union (EU) law. The authorization and regulation of GMOs in the EU are shared between national authorities and the European Commission; however, the criteria for their evaluation are defined in the EU regulatory framework. The use of GMOs is governed by two key directives, depending on the context: Directive 2009/41/EC regulates the use of GMOs in contained environments such as laboratories, hospitals, or industrial facilities, while Directive 2001/18/EC and Directive (EU) 2018/350 govern their deliberate release into the environment and commercialization (Dederer and Hamburger, 2022; Broll et al., 2025).

## 4 Opportunities for circular economy in bauxite residue valorization

The circular economy paradigm seeks to transform the traditional “take-make-waste” linear model into a regenerative system that decouples economic growth from resource consumption. In the alumina industry, particularly regarding legacy bauxite residue storage, this approach is embodied by three core principles: eliminating waste and pollution, circulating products and materials, and regenerating natural ecosystems (Ellen MacArthur Foundation, 2013). When applying these principles, bauxite residue can be valorized through integrated pathways that combine its value-added utilization with conventional extraction and innovative bioleaching, maximizing the recovery of valuable elements while enhancing sustainability and economic viability.

**FIGURE 11:**
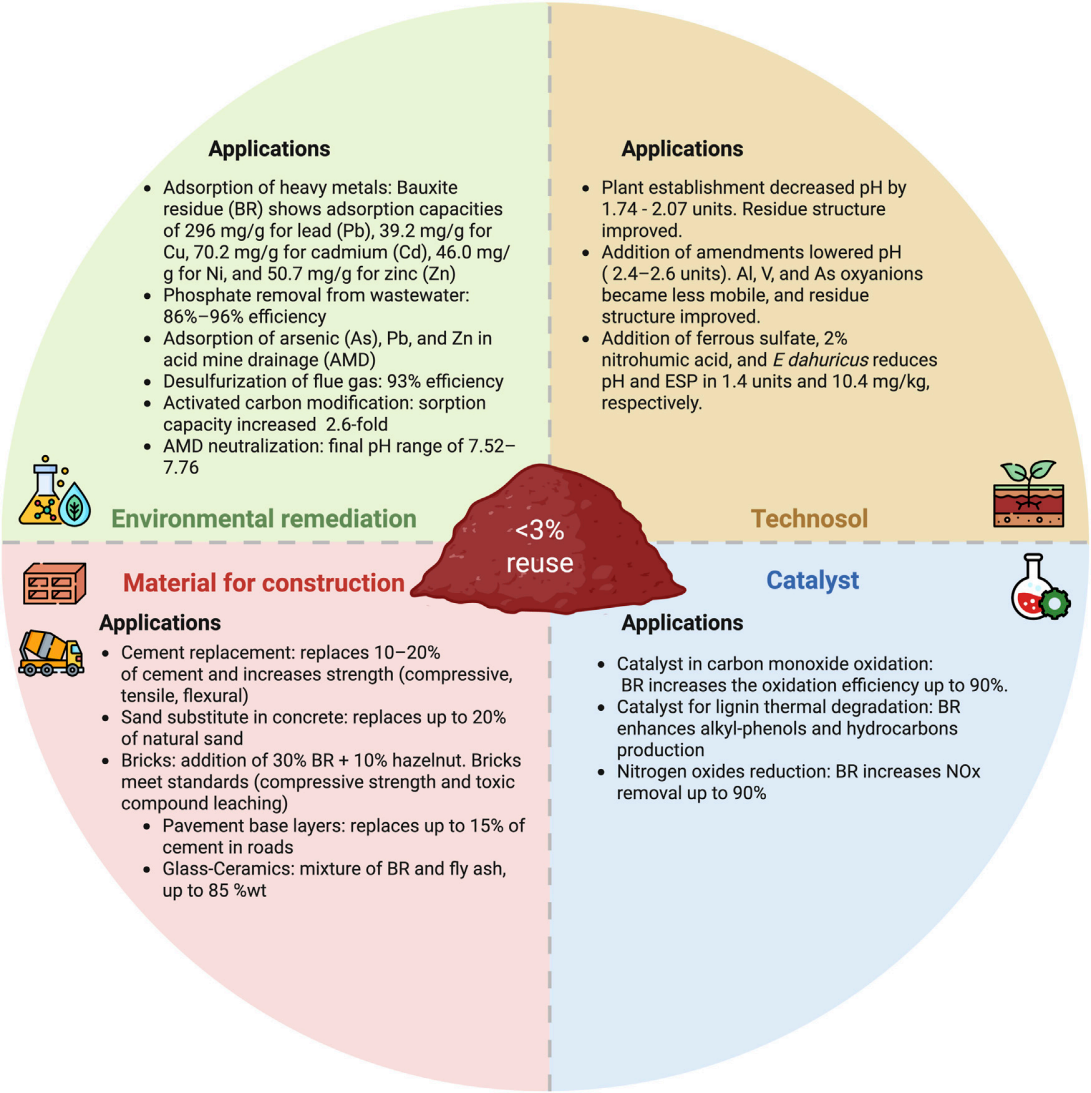
Examples of uses of bauxite residue as a construction materials (Yang et al., 2008b; Metilda et al., 2015; Atan et al., 2021; Viyasun et al., 2021; Salim et al., 2023; Raj et al., 2024; Kusumanjali et al., 2025), for environmental remediation (Bertocchi et al., 2006; Huang et al., 2008; Couperthwaite et al., 2012; Tao et al., 2019; Yang et al., 2020; Smičiklas et al., 2021; Kyrii et al., 2023; Liu et al., 2023; Gauthier et al., 2024; Liu et al., 2024), as a catalyst (Sushil and Batra, 2008; Sushil and Batra, 2012; Wang et al., 2018; Chen et al., 2023; Li Y. et al., 2024; Xu et al., 2024) and technosol formation (Bray et al., 2018; Hu et al., 2024; Lu et al., 2025).

Given the broad spectrum of recoverable elements and challenges linked to the alkalinity and contaminant content of bauxite residue, strategies that simultaneously address environmental constraints, resource recovery, and post-extraction utilization are essential. Value-added applications, such as construction materials, environmental remediation agents, catalysts, and transformation into technosol (Figure 11), alongside metal recovery, constitute the two main pillars for sustainable reuse of alumina refinery residue. By coupling value-added applications of bauxite residue with sustainable processes for critical metal recovery, this integrated framework repositions bauxite residue from an environmental liability to a regenerative resource.

Furthermore, industrial symbiosis can be integrated into the bioprocessing system (Liu et al., 2009a) through the use of organic acid-producing microorganisms capable of converting agro-industrial residues into substrates for bioleaching agent synthesis. This approach reduces costs associated with critical mineral extraction and alumina refinery waste revalorization, as pure carbon sources like glucose can account for up to 44% of total bioleaching process costs (Thompson et al., 2017). Various fungal species extracellularly produce cellulolytic enzymes that degrade lignocellulosic biomass into simpler sugars. Agricultural by-products such as sugarcane bagasse, faba bean straw, and wheat straw, have been successfully employed for organic acid production (Egbe et al., 2022; Mahgoub et al., 2022). For instance, bioleaching of Al from bauxite residue using *P*. *simplicissimum* and low-cost molasses as the carbon source at 1% w/v pulp density achieved 86.6% metal recovery (Shah et al., 2022).

Most bacterial species require pretreatment of lignocellulosic biomass to convert polysaccharides into fermentable sugars, which are then converted into organic acids. In particular, *G*. *oxydans*, a widely studied bioleaching species, has been used to produce gluconic acid from various cellulosic hydrolysates (Zhou and Xu, 2019; Dai et al., 2022). This species has also been applied to leach REEs from industrial solid waste using hydrolyzed potato wastewater, achieving leaching recoveries comparable to those obtained with glucose as the carbon source (25.7% and 25.1%, respectively) (Jin et al., 2019). By incorporating agricultural waste into the bioprocessing chain, reliance on costly pure carbon sources such as glucose is reduced, lowering operational costs and embedding nutrient recycling within the system.

**FIGURE 12:**
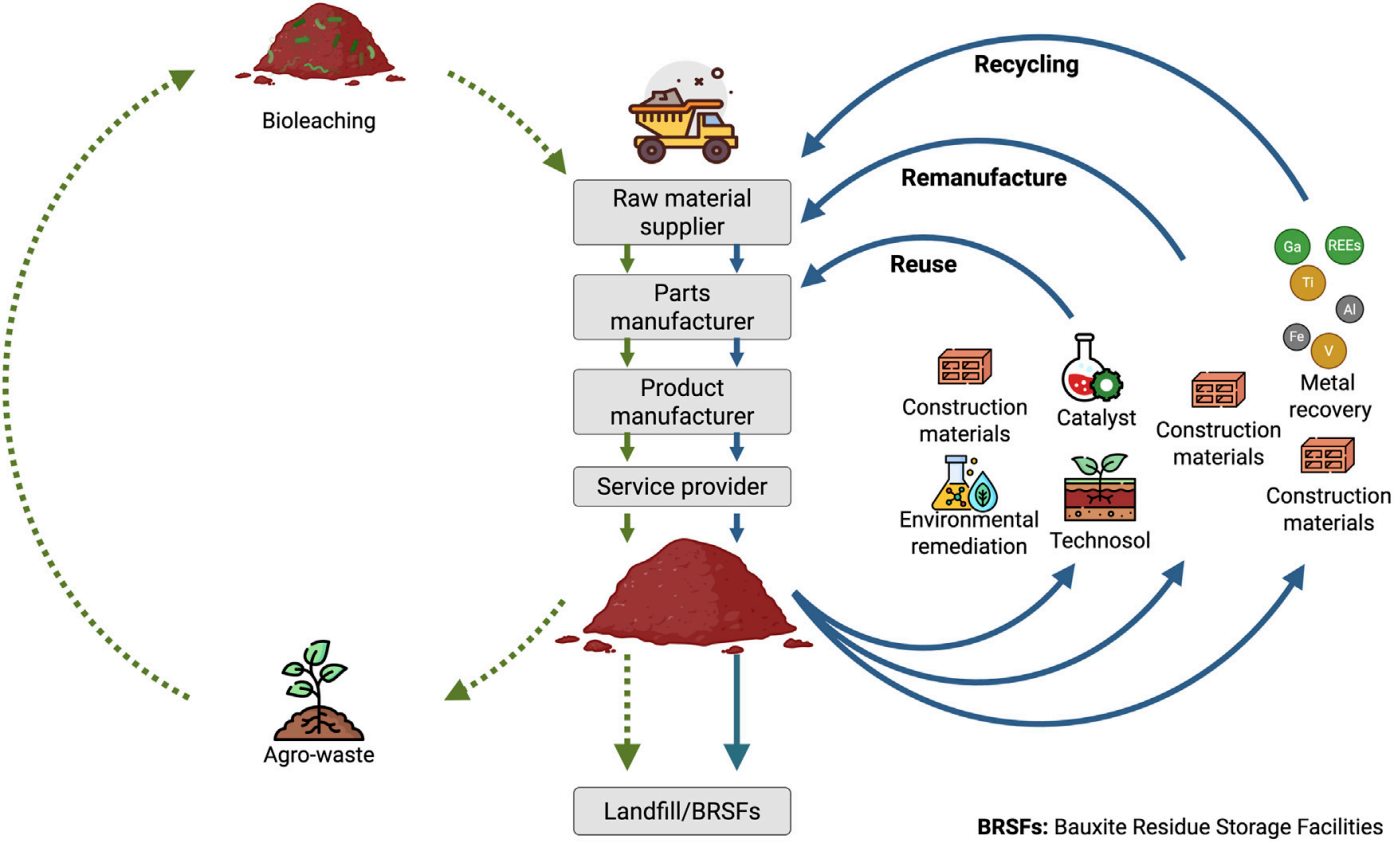
Butterfly diagram illustrating the integration of bioprocessing and valorization of bauxite residue prior to its disposal in bauxite residue storage facilities (BRSFs). The technical cycle is indicated by blue arrows, whereas the biological cycle is depicted with green dotted arrows. Adapted from (Ellen MacArthur Foundation, 2013).

The integration of circular economy principles within the alumina refinery sector can be conceptualized through a butterfly diagram (Figure 12). Within the technical cycle illustrated in the diagram, the recycling loop represents the recovery of high-value products from bauxite residue. This process promotes material circularity and provides a secondary supply of critical raw materials essential for clean energy technologies. In contrast, waste minimization is exemplified by the remanufacturing of bauxite residue into construction materials, as well as by its application in environmental remediation, catalyst production, and technosol formation. These pathways collectively constitute the reuse loop within the technical cycle, wherein the inherent physicochemical properties and composition of the residue are utilized with minimal or no additional processing. Together, the recycling and reuse loops function as complementary components of the technical cycle, jointly enhancing resource efficiency and supporting material sustainability. The proposed industrial symbiosis, in which organic waste streams, particularly agro-industrial residues, are employed as substrates to generate high-value products, such as bioleaching agents, through microbial fermentation, is represented in the biological cycle. This approach exemplifies industrial symbiosis, whereby by-products from one sector (agriculture) serve as inputs for another (mining and metal recovery), thereby promoting the sustainable use of natural resources and enhancing both overall system sustainability and the valorization of alumina refinery residue (Ellen MacArthur Foundation, 2013).

## 5 Conclusion and future perspectives

Bauxite residue presents both environmental and safety challenges for communities near disposal sites, while simultaneously representing a considerable secondary resource of critical minerals such as REEs, V, Ga, and Ti, essential for the development of cleaner and more sustainable technologies, with global demand increasing annually. However, current extraction methods are energy-intensive and generate substantial amounts of hazardous waste. Therefore, there is a pressing need to develop extraction processes that are not only environmentally sustainable but also scalable and industrially feasible, ensuring a balance between resource utilization and environmental protection. Bioleaching offers a promising potential to extract critical metals by employing high organic acid-producing microbial species. Although the potential of bauxite residue as a secondary source of critical metals is widely recognized, industrial-scale recovery has not yet been achieved. Recent research has primarily focused on the direct application of microorganisms, rather than on optimizing bioleaching processes aided by modelling, machine learning and artificial intelligence-assisted approaches or by improving microbial performance through strain optimization. One underexplored but promising approach for the latter is ALE, which can be used to enhance microbial tolerance to metals in solution and to bauxite residue, thereby increasing the volume of waste that can be treated. This strategy could be applied to optimize strains already used in industry, improving their resistance to bauxite residue by enhancing both their ability to tolerate the presence of solids and the toxic components present or leached, as well as their capacity to produce high levels of organic acids, thereby contributing to improved metal recovery and the valorization of alumina refinery waste. Additionally, the genetic engineering of microbial strains for more efficient production of bioleaching agents represents a key strategy for improving carbon source utilization and overall bioleaching performance, however, its applicability must be evaluated in accordance with the legislation of each country. Altogether, these strategies present promising opportunities for recovering and reusing critical metals from alumina refinery waste, not only as an environmental necessity but also as a strategic step toward securing a sustainable and resilient supply of materials essential for the green energy transition. Finally, integrating industrial processes with circular economy principles is vital for establishing an economically viable and environmentally sustainable valorization pathway for bauxite residue.

## References

[B1] Aaseth J. Berlinger B. (2022). “Lanthanum,” in Handbook on the toxicology of metals. Editors Nordberg G. F. Costa M. 5 ed (London, UK: Elsevier), 419–425.

[B2] Abedi E. Mohammad S. Hashemi B. (2020). Lactic acid production - producing microorganisms and substrates sources-state of art. Heliyon 6 (10), e04974. 10.1016/j.heliyon.2020.e04974 33088933 PMC7566098

[B3] Abhilash Hedrich S. Schippers A. (2021). Distribution of scandium in red mud and extraction using *Gluconobacter oxydans* . Hydrometallurgy 202 (5), 105621. 10.1016/j.hydromet.2021.105621

[B4] Agrawal S. Dhawan N. (2021). Evaluation of red mud as a polymetallic source - a review. Miner. Eng. 171, 107084. 10.1016/j.mineng.2021.107084

[B5] Ai C. McCarthy S. Eckrich V. Rudrappa D. Qiu G. Blum P. (2016). Increased acid resistance of the archaeon, *Metallosphaera sedula* by adaptive laboratory evolution. J. Industrial Microbiol. Biotechnol. 43 (10), 1455–1465. 10.1007/s10295-016-1812-0 27520549

[B6] Alam S. Das B. K. Das S. K. (2018). Dispersion and sedimentation characteristics of red mud. J. Hazard. Toxic, Radioact. Waste 22 (4), 04018025. 10.1061/(asce)hz.2153-5515.0000420

[B7] Amenaghawon A. N. Ayere J. E. Amune U. O. Otuya I. C. Abuga E. C. Anyalewechi C. L. (2024). A comprehensive review of recent advances in the applications and biosynthesis of oxalic acid from bio-derived substrates. Environ. Res. 251, 118703. 10.1016/j.envres.2024.118703 38518912

[B8] Amiri F. Yaghmaei S. Mousavi S. M. Sheibani S. (2011). Recovery of metals from spent refinery hydrocracking catalyst using adapted *Aspergillus niger* . Hydrometallurgy 109 (1), 65–71. 10.1016/j.hydromet.2011.05.008

[B9] Arshadi M. Esmaeili A. Yaghmaei S. (2020). Investigating critical parameters for bioremoval of heavy metals from computer printed circuit boards using the fungus *Aspergillus niger* . Hydrometallurgy 197, 105464. 10.1016/j.hydromet.2020.105464

[B10] Atan E. Sutcu M. Cam A. S. (2021). Combined effects of Bayer process bauxite waste (red mud) and agricultural waste on technological properties of fired clay bricks. J. Build. Eng. 43, 103194. 10.1016/j.jobe.2021.103194

[B11] Aung K. M. Ting Y. P. (2005). Bioleaching of spent fluid catalytic cracking catalyst using *Aspergillus niger* . J. Biotechnol. 116 (2), 159–170. 10.1016/j.jbiotec.2004.10.008 15664080

[B12] Aung H. Y. Boyarintsev A. Stepanov S. Shoustikov A. (2021). Current key options for management of industrial alkaline waste of alumina production (red mud). E3S Web Conf. 284, 01003. 10.1051/e3sconf/202128401003

[B13] Australian Government (2024a). Federal register of legislation - gene technology regulations 2001. Available online at: https://www.legislation.gov.au/F2001B00162/latest/text (Accessed July 20, 2025).

[B14] Australian Government (2024b). Gene technology act 2000. Available online at: https://www.legislation.gov.au/C2004A00762 (Accessed July 20, 2025).

[B15] Australian Government Department of Health (2024). “Disability and ageing - office of the gene technology regulator,” in Australia's gene technology regulatory system. Available online at: https://www.ogtr.gov.au/about-ogtr/australias-gene-technology-regulatory-system (Accessed July 20, 2025).

[B16] Azimi Y. Hosseini M. R. Azimi E. Pedram H. (2024). Comparison of enhanced neural network and response surface models in predicting bio-dissolution of aluminum and vanadium from bauxite residue by isolated *Aspergillus niger* strains. J. Taiwan Inst. Chem. Eng. 164, 105685. 10.1016/j.jtice.2024.105685

[B17] Bahaloo-Horeh N. Mousavi S. M. Baniasadi M. (2018). Use of adapted metal tolerant *Aspergillus niger* to enhance bioleaching efficiency of valuable metals from spent lithium-ion mobile phone batteries. J. Clean. Prod. 197, 1546–1557. 10.1016/j.jclepro.2018.06.299

[B18] Balaram V. (2019). Rare earth elements: a review of applications, occurrence, exploration, analysis, recycling, and environmental impact. Geosci. Front. 10 (4), 1285–1303. 10.1016/j.gsf.2018.12.005

[B19] Balomnenos E. Kastritis D. Panias D. Paspaliaris I. Boufounos D. (2016). “The enexal bauxite residue treatment process: industrial-scale pilot plant results,” in Light metals 2014. Editor Grandfield J. (Cham, Switzerland: Springer), 143–147.

[B20] Barreiro C. Martín J. F. García-Estrada C. (2012). Proteomics shows new faces for the old penicillin producer *Penicillium chrysogenum* . J. Biomed. Biotechnol. 2012, 105109. 10.1155/2012/105109 22318718 PMC3270403

[B21] Bertocchi A. F. Ghiani M. Peretti R. Zucca A. (2006). Red mud and fly ash for remediation of mine sites contaminated with As, Cd, Cu, Pb and Zn. J. Hazard. Mater. 134 (1), 112–119. 10.1016/j.jhazmat.2005.10.043 16326004

[B22] Binnemans K. Jones P. T. Blanpain B. Gerven T. V. Pontikes Y. (2015). Towards zero-waste valorisation of rare-earth-containing industrial process residues: a critical review. J. Clean. Prod. 99, 17–38. 10.1016/j.jclepro.2015.02.089

[B23] Biswal B. K. Balasubramanian R. (2023). Recovery of valuable metals from spent lithium-ion batteries using microbial agents for bioleaching: a review. Front. Microbiol. 14, 1197081. 10.3389/fmicb.2023.1197081 37323903 PMC10264615

[B24] Borra C. R. Pontikes Y. Binnemans K. Gerven T. V. (2015). Leaching of rare earths from bauxite residue (red mud). Miner. Eng. 76, 20–27. 10.1016/j.mineng.2015.01.005

[B25] Bray A. W. Stewart D. I. Courtney R. Rout S. P. Humphreys P. N. Mayes W. M. (2018). Sustained bauxite residue rehabilitation with gypsum and organic matter 16 years after initial treatment. Environ. Sci. and Technol. 52 (1), 152–161. 10.1021/acs.est.7b03568 29182867

[B26] Bringer S. Bott M. (2016). “Central carbon metabolism and respiration in *Gluconobacter oxydans* ,” in Acetic acid bacteria: ecology and physiology. Editors Matsushita K. Toyama H. Tonouchi N. Okamoto-Kainuma A. (Tokyo, Japan: Springer), 235–253.

[B27] Broll H. Bendiek J. Braeuning A. Eckermann K. N. Gebhardt A. Grohmann L. (2025). Current status and trends in the analysis of GMO and new genomic techniques. J. Consumer Prot. Food Saf. 20 (1), 89–92. 10.1007/s00003-025-01542-y

[B28] Burgstaller W. Schinner F. (1993). Leaching of metals with fungi. J. Biotechnol. 27 (2), 91–116. 10.1016/0168-1656(93)90101-r

[B29] Burke I. T. Mayes W. M. Peacock C. L. Brown A. P. Jarvis A. P. Gruiz K. (2012). Speciation of arsenic, chromium, and vanadium in red mud samples from the Ajka spill site, Hungary. Environ. Sci. and Technol. 46 (6), 3085–3092. 10.1021/es3003475 22324637

[B30] Burlacu A. Israel-Roming F. Cornea C. P. (2017). Fungal strains improvement for xylanase over production through physical and chemical mutagenesis. AgroLife Sci. J. 6 (1), 40–47.

[B31] Butcher T. Brown T. (2014). “Gallium,” in Critical metals handbook. Editor Gunn G. (Chichester, UK: John Wiley and Sons, Ltd), 150–176.

[B32] Cablik V. (2007). Characterization and applications of red mud from bauxite processing. Gospod. Surowcami Mineralnymi-Mineral Resour. Manag. 23 (4), 27–38.

[B33] Cao W. Yan L. Li M. Liu X. Xu Y. Xie Z. (2020). Identification and engineering a C4-dicarboxylate transporter for improvement of malic acid production in *Aspergillus niger* . Appl. Microbiol. Biotechnol. 104 (22), 9773–9783. 10.1007/s00253-020-10932-1 32997202

[B34] Chen J. Liu Y. Diep P. Mahadevan R. (2022). Harnessing synthetic biology for sustainable biomining with Fe/S-oxidizing microbes. Front. Bioeng. Biotechnol. 10, 920639. 10.3389/fbioe.2022.920639 36131722 PMC9483119

[B35] Chen J. Wang Y. Liu Z. (2023). Red mud-based catalysts for the catalytic removal of typical air pollutants: a review. J. Environ. Sci. 127, 628–640. 10.1016/j.jes.2022.06.027 36522092

[B36] Chernoburova O. Chagnes A. (2023). “Processing and extraction of critical raw materials from residues,” in Mining and processing residues. Editors Chernoburova O. Chagnes A. (Amsterdam, Netherlands: Elsevier), 71–183.

[B37] Couperthwaite S. J. Johnstone D. W. Millar G. J. Frost R. L. (2012). Neutralization of acid sulfate solutions using bauxite refinery residues and its derivatives. Industrial and Eng. Chem. Res. 52 (4), 1388–1395. 10.1021/ie301618p

[B38] Couturier J. Levard C. Collin B. Chaurand P. Vidal V. Mathon O. (2025). Dissolution of rare earth elements: exploring the ability of deep eutectic solvents and organic acid solutions, the case of lactic acid. Sep. Purif. Technol. 366, 132740. 10.1016/j.seppur.2025.132740

[B39] Cozzolino A. Cappai G. Cara S. Muñoz J. A. Milia S. Tamburini E. (2024). Bioleaching of secondary and critical raw materials from red mud by a mixed culture in a semi-continuous reactor. Hydrometallurgy 224, 106263. 10.1016/j.hydromet.2024.106263

[B40] Crundwell F. K. (2003). How do bacteria interact with minerals? Hydrometallurgy 71 (1), 75–81. 10.1016/s0304-386x(03)00175-0

[B41] Dai L. Lian Z. Zhang R. Nawaz A. Ul-Haq I. Zhou X. (2022). Multi-strategy in production of high titer gluconic acid by the fermentation of concentrated cellulosic hydrolysate with *Gluconobacter oxydans* . Industrial Crops Prod. 189, 115748. 10.1016/j.indcrop.2022.115748

[B42] De Jesus A. Mendonça S. (2018). Lost in transition? Drivers and barriers in the eco-innovation road to the circular economy. Ecol. Econ. 145, 75–89. 10.1016/j.ecolecon.2017.08.001

[B43] De Vuyst L. Van Kerrebroeck S. Leroy F. (2017). “Microbial ecology and process technology of sourdough fermentation,” in Advances in applied microbiology. Editors Sariaslani S. Gadd G. M. (London, UK: Academic Press), 49–160. 10.1016/bs.aambs.2017.02.003 28732554

[B44] Dederer H. G. Hamburger D. (2022). Are genome-edited micro-organisms covered by directive 2009/41/EC? - implications of the CJEU’s judgment in the case C-528/16 for the contained use of genome-edited micro-organisms. J. Law Biosci. 9 (1), lsab033. 10.1093/jlb/lsab033 35106181 PMC8801222

[B45] Desroches S. Daydé S. Berthon G. (2000). Aluminum speciation studies in biological fluids. Part 6. Quantitative investigation of aluminum(III)–tartrate complex equilibria and their potential implications for aluminum metabolism and toxicity. J. Inorg. Biochem. 81 (4), 301–312. 10.1016/s0162-0134(00)00072-6 11065194

[B46] Dezam A. P. G. Vasconcellos V. M. Lacava P. T. Farinas C. S. (2017). Microbial production of organic acids by endophytic fungi. Biocatal. Agric. Biotechnol. 11, 282–287. 10.1016/j.bcab.2017.08.001

[B47] Dong Y. Zan J. Lin H. (2023). Bioleaching of heavy metals from metal tailings utilizing bacteria and fungi: mechanisms, strengthen measures, and development prospect. J. Environ. Manag. 344, 118511. 10.1016/j.jenvman.2023.118511 37418918

[B48] Drábek O. Kipkoech Kiplagat I. Komárek M. Tejnecký V. Borůvka L. (2015). Study of interactions between relevant organic acids and aluminium in model solutions using HPLC and IC. Soil Water Res. 10 (3), 172–180. 10.17221/256/2014-swr

[B49] Dragosits M. Mattanovich D. (2013). Adaptive laboratory evolution - principles and applications for biotechnology. Microb. Cell Factories 12 (1), 64. 10.1186/1475-2859-12-64 23815749 PMC3716822

[B50] Dürig J. Calcagni M. Buschmann J. (2023). Transition metals in angiogenesis - a narrative review. Mater. Today Bio 22, 100757. 10.1016/j.mtbio.2023.100757 37593220 PMC10430620

[B51] Dusengemungu L. Kasali G. Gwanama C. Mubemba B. (2021). Overview of fungal bioleaching of metals. Environ. Adv. 5, 100083. 10.1016/j.envadv.2021.100083

[B52] Edward C. J. Kotsiopoulos A. Harrison S. T. L. (2018). Low-level thiocyanate concentrations impact on iron oxidation activity and growth of *Leptospirillum ferriphilum* through inhibition and adaptation. Res. Microbiol. 169 (10), 576–581. 10.1016/j.resmic.2018.10.003 30391481

[B53] Egbe N. E. Ihediwa L. Abdulsalami M. S. Adebayo A. (2022). Citric acid production from agricultural wastes using *Aspergillus niger* isolated from some locations within Kaduna metropolis, Nigeria. J. Appl. Sci. Environ. Manag. 26 (9), 1607–1614. 10.4314/jasem.v26i9.22

[B54] Ellen MacArthur Foundation (2013). Towards the circular economy vol. 1: an economic and business rationale for an accelerated transition. Isle of Wight, United Kingdom: Ellen MacArthur Foundation.

[B55] European Commission Directorate-General for Internal Market, Industry, Entrepreneurship SMEs (2023). Study on the critical raw materials for the EU 2023 - final report. Luxemburg: Publications Office of the European Union. doi/10.2873/725585

[B56] Fang C. Lou R. Ju Y. Jia Y. Wu J. Chen Y. (2025). Critical metal recovery from red mud: a systematic review of sustainable extraction technologies and circular economy potential. J. Environ. Chem. Eng. 13 (5), 118985. 10.1016/j.jece.2025.118985

[B57] Feigl V. Medgyes-Horváth A. Kari A. Török Á. Bombolya N. Berkl Z. (2024). The potential of Hungarian bauxite residue isolates for biotechnological applications. Biotechnol. Rep. 41, e00825. 10.1016/j.btre.2023.e00825 38225962 PMC10788403

[B58] Feng S. Yang H. Wang W. (2015). Microbial community succession mechanism coupling with adaptive evolution of adsorption performance in chalcopyrite bioleaching. Bioresour. Technol. 191, 37–44. 10.1016/j.biortech.2015.04.122 25978855

[B59] Findlow J. A. Duffield J. R. Williams D. R. (1990). The chemical speciation of aluminium in milk. Chem. Speciat. and Bioavailab. 2 (1), 3–32. 10.1080/09542299.1990.11083122

[B60] Foley N. K. Jaskula B. W. Kimball B. E. Schulte R. F. (2017). “Gallium,” in Critical mineral resources of the United States - economic and environmental geology and prospects for future supply: U.S. geological survey professional paper 1802. Editors Schulz K. J. DeYoung J. Seal R. R. Bradley D. (Reston, VA: U. S. Geological Survey), H1–H35.

[B61] Food and Drug Administration (FDA), U.S (2024). Agricultural biotechnology. Available online at: https://www.fda.gov/food/consumers/agricultural-biotechnology (Accessed July 20, 2025).

[B62] Gad S. C. (2024). “Cerium,” in Encyclopedia of toxicology. Editor Wexler P. 4 ed (Oxford, UK: Academic Press), 711–714.

[B63] Gadd G. M. (1994). “Interactions of fungi with toxic metals,” in The genus *aspergillus*: from taxonomy and genetics to industrial application. Editors Powell K. A. Renwick A. Peberdy J. F. (New York, NY: Springer), 361–374.

[B64] Gadd G. M. (1999). “Fungal production of citric and oxalic acid: importance in metal speciation, physiology, and biogeochemical processes,” in Advances in microbial physiology. Editor Poole R. K. (London, UK: Academic Press), 47–92. 10.1016/s0065-2911(08)60165-4 10500844

[B65] Gamaletsos P. N. Godelitsas A. Kasama T. Kuzmin A. Lagos M. Mertzimekis T. J. (2016). The role of nano-perovskite in the negligible thorium release in seawater from Greek bauxite residue (red mud). Sci. Rep. 6 (1), 21737. 10.1038/srep21737 26899139 PMC4761986

[B66] Gao W. Sun Z. Cao H. Ding H. Zeng Y. Ning P. (2020). Economic evaluation of typical metal production process: a case study of vanadium oxide production in China. J. Clean. Prod. 256, 120217. 10.1016/j.jclepro.2020.120217

[B67] Gauthier A. Omana B. Amin F. Le Coustumer P. (2024). Waste bauxite residue valorization as trace metal sorbent: application to acid mine drainage remediation. Water 16 (22), 3255. 10.3390/w16223255

[B68] Geoscience Australia (2024a). Critical minerals and their uses. Available online at: https://www.ga.gov.au/scientific-topics/minerals/critical-minerals/critical-minerals-and-their-uses (Accessed July 20, 2025).

[B69] Geoscience Australia (2024b). Critical minerals at geoscience Australia. Available online at: https://www.ga.gov.au/scientific-topics/minerals/critical-minerals (Accessed July 20, 2025).

[B70] Ghorbani Y. Oliazadeh M. Shahvedi A. (2008). Aluminum solubilization from red mud by some indigenous fungi in Iran. J. Appl. Biosci. 7, 207–213.

[B71] Gnanasekaran R. Petchiammal A. Subhashree B. D. Anubha M. Dinakarkumar Y. (2022). Synthesis of citric acid using novel *Aspergillus niveus* obtained from agricultural wastes. Ann. Adv. Chem. 6 (1), 51–55. 10.29328/journal.aac.1001032

[B72] Goronovski A. Rivera R. M. Gerven T. V. Tkaczyk A. H. (2021). Radiological assessment of bauxite residue processing to enable zero-waste valorisation and regulatory compliance. J. Clean. Prod. 294, 125165. 10.1016/j.jclepro.2020.125165

[B73] Gräfe M. Landers M. Tappero R. Austin P. Gan B. Grabsch A. (2011a). Combined application of QEM‐SEM and hard X‐ray microscopy to determine mineralogical associations and chemical speciation of trace metals. J. Environ. Qual. 40 (3), 767–783. 10.2134/jeq2010.0214 21546662

[B74] Gräfe M. Power G. Klauber C. (2011b). Bauxite residue issues: III. Alkalinity and associated chemistry. Hydrometallurgy 108 (1), 60–79. 10.1016/j.hydromet.2011.02.004

[B75] Grąz M. (2024). Role of oxalic acid in fungal and bacterial metabolism and its biotechnological potential. World J. Microbiol. Biotechnol. 40 (6), 178. 10.1007/s11274-024-03973-5 38662173 PMC11045627

[B76] Gu H. Li W. Li Z. Guo T. Wen H. Wang N. (2020). Leaching behavior of lithium from bauxite residue using acetic acid. Min. Metallurgy and Explor. 37 (2), 443–451. 10.1007/s42461-020-00181-1

[B77] Guo W. He R. Ma L. Jia W. Li D. Chen S. (2014). Construction of a constitutively expressed homo-fermentative pathway in *Lactobacillus brevis* . Appl. Microbiol. Biotechnol. 98 (15), 6641–6650. 10.1007/s00253-014-5703-x 24728715

[B78] Hamel R. Levasseur R. Appanna V. D. (1999). Oxalic acid production and aluminum tolerance in *Pseudomonas fluorescens* . J. Inorg. Biochem. 76 (2), 99–104. 10.1016/s0162-0134(99)00120-8 10612061

[B79] Han Z. Gao B. Cheng H. Zhou H. Wang Y. Chen Z. (2024). *Lactobacillus pentosus* enabled bioleaching of red mud at high pulp density and simultaneous production of lactic acid without supplementation of neutralizers. J. Environ. Chem. Eng. 12 (6), 114650. 10.1016/j.jece.2024.114650

[B80] Hanke T. Nöh K. Noack S. Polen T. Bringer S. Sahm H. (2013). Combined fluxomics and transcriptomics analysis of glucose catabolism via a partially cyclic pentose phosphate pathway in *Gluconobacter oxydans* 621H. Appl. Environ. Microbiol. 79 (7), 2336–2348. 10.1128/aem.03414-12 23377928 PMC3623255

[B81] Harmaji A. Jafari R. Simard G. (2024). Valorization of residue from aluminum industries: a review. Materials 17 (21), 5152. 10.3390/ma17215152 39517428 PMC11546596

[B82] Harrison S. T. L. Broadhurst J. L. Opitz A. Fundikwa B. Stander H. M. Mostert L. (2020). An industrial ecology approach to sulphide-containing mineral wastes to minimise ARD formation. Water Res. Comm.

[B83] Healy S. (2022). Sustainable bauxite residue management guidance. Int. Alum. Inst. (IAI). Available online at: https://international-aluminium.org/wp-content/uploads/2022/04/BRManagementGuidance.pdf (Accessed July 20, 2025).

[B84] Hena S. bt Abdullah N. F. Keong L. C. Mohamed Najar P. A. Gutierrez L. Croué J. P. (2022). Zero residual heavy metals in aqueous media using composite coagulant converted from bauxite residue. Int. J. Environ. Sci. Technol. 20 (5), 5453–5470. 10.1007/s13762-022-04336-z

[B85] Higgins D. Curtin T. Pawlett M. Courtney R. (2016). The potential for constructed wetlands to treat alkaline bauxite-residue leachate: *phragmites australis* growth. Environ. Sci. Pollut. Res. 23 (23), 24305–24315. 10.1007/s11356-016-7702-1 27655613

[B86] Hirasawa T. Maeda T. (2022). Adaptive laboratory evolution of microorganisms: methodology and application for bioproduction. Microorganisms 11 (1), 92. 10.3390/microorganisms11010092 36677384 PMC9864036

[B87] Hronská H. Micháliková S. Rosenberg M. (2017). Microbial production of specialty C4 dicarboxylic acids from maleic anhydride. J. Food Nutr. Res. 56 (3), 219–231.

[B88] Hu L. Du P. Ren J. Zhang Y. Liu Y. Chen K. (2024). Restoration-mediated protein substances preferentially drive underlying bauxite residue macroaggregate formation during the simulated ecological reconstruction process. Sci. Total Environ. 951, 175636. 10.1016/j.scitotenv.2024.175636 39168338

[B89] Huang W. Wang S. Zhu Z. Li L. Yao X. Rudolph V. (2008). Phosphate removal from wastewater using red mud. J. Hazard. Mater. 158 (1), 35–42. 10.1016/j.jhazmat.2008.01.061 18314264

[B90] Huang S. Liu R. Sun M. Li X. Guan Y. Lian B. (2022). Transcriptome expression analysis of the gene regulation mechanism of bacterial mineralization tolerance to high concentrations of Cd^2+^ . Sci. Total Environ. 806, 150911. 10.1016/j.scitotenv.2021.150911 34653453

[B91] Ilkhani Z. Vakilchap F. Sadeghi N. Mohammad Mousavi S. (2024). Base metals (Fe, Al, Ti) and rare earth elements (Ce, La, Pr) leaching from red mud through an efficient chemical-biological hybrid approach. Miner. Eng. 208, 108603. 10.1016/j.mineng.2024.108603

[B92] Ilyas S. Kim H. Srivastava R. R. (2021). Feasibility of the bio-mobilization of rare earth elements from bauxite residual red mud. Environ. Sci. Proc. 6 (1), 5. 10.3390/iecms2021-09334

[B93] Irfan M. Syed Q. Javed J. (2011). UV mutagenesis of *Aspergillus niger* for enzyme production in submerged fermentation. Pak. J. Biochem. and Mol. Biol. 44 (4), 137–140.

[B94] Izcapa-Treviño C. Loera O. Tomasini-Campocosio A. Esparza-García F. Salazar-Montoya J. A. Díaz-Cervantes M. D. (2009). Fenton (H_2_O_2_/Fe) reaction involved in *Penicillium* sp. culture for DDT [1,1,1-trichloro-2,2-bis(p-chlorophenyl)ethane] degradation. J. Environ. Sci. Health 44 (8), 798–804. 10.1080/03601230903238368 20183092

[B95] Jahan K. Supty M. S. A. Lee J. S. Choi K. H. (2024). Transcriptomic analysis provides new insights into the tolerance mechanisms of green macroalgae *Ulva prolifera* to high temperature and light stress. Biology 13 (9), 725. 10.3390/biology13090725 39336152 PMC11428574

[B96] Janusz W. Pikus S. Skwarek E. Olszewska E. (2020). Synthesis of citrates of selected lanthanides (Er, Ho and Lu). Physicochem. Problems Mineral Process. 56 (6), 225–234. 10.37190/ppmp/128739

[B97] Jiang Y. Qin X. Zhu F. Zhang Y. Zhang X. Hartley W. (2023). Halving gypsum dose by *Penicillium oxalicum* on alkaline neutralization and microbial community reconstruction in bauxite residue. Chem. Eng. J. 451, 139008. 10.1016/j.cej.2022.139008

[B98] Jin H. Reed D. W. Thompson V. S. Fujita Y. Jiao Y. Crain-Zamora M. (2019). Sustainable bioleaching of rare earth elements from industrial waste materials using agricultural wastes. ACS Sustain. Chem. and Eng. 7 (18), 15311–15319. 10.1021/acssuschemeng.9b02584

[B99] Jung H. Inaba Y. Banta S. (2022). Genetic engineering of the acidophilic chemolithoautotroph *Acidithiobacillus ferrooxidans* . Trends Biotechnol. 40 (6), 677–692. 10.1016/j.tibtech.2021.10.004 34794837

[B100] Jung H. Inaba Y. Banta S. (2024). CRISPR/dCas12a knock-down of *Acidithiobacillus ferrooxidans* electron transport chain bc_1_ complexes enables enhanced metal sulfide bioleaching. J. Biol. Chem. 300 (9), 107703. 10.1016/j.jbc.2024.107703 39173952 PMC11421330

[B101] Kalmykova Y. Sadagopan M. Rosado L. (2018). Circular economy - from review of theories and practices to development of implementation tools. Resour. Conservation Recycl. 135, 190–201. 10.1016/j.resconrec.2017.10.034

[B102] Kannan P. Banat F. Hasan S. W. Abu Haija M. (2021). Neutralization of Bayer bauxite residue (red mud) by various brines: a review of chemistry and engineering processes. Hydrometallurgy 206, 105758. 10.1016/j.hydromet.2021.105758

[B103] Karanam S. K. Medicherla N. R. (2008). Enhanced lipase production by mutation induced *Aspergillus japonicus* . Afr. J. Biotechnol. 7 (12), 2064–2067. 10.5897/AJB2008.000-5054

[B104] Katsumata K. I. Ohno Y. Tomita K. Sakai M. Nakajima A. Kakihana M. (2011). Preparation of TiO_2_ thin films using water-soluble titanium complexes and their photoinduced properties. Photochem. Photobiol. 87 (5), 988–994. 10.1111/J.1751-1097.2011.00944.X 21615742

[B105] Kehagia F. (2014). Construction of an unpaved road using industrial by-products *(bauxite residue)* . WSEAS transactions on environment and development, 10, 160–168.

[B106] Khandelwal R. Srivastava P. Bisaria V. S. (2023). Recent advances in the production of malic acid by native fungi and engineered microbes. World J. Microbiol. Biotechnol. 39 (8), 217. 10.1007/s11274-023-03666-5 37269376

[B107] Kim J. Kim D. G. Ryu K. H. (2023). Enhancing response surface methodology through coefficient clipping based on prior knowledge. Processes 11 (12), 3392. 10.3390/pr11123392

[B108] Kiskira K. Lymperopoulou T. Tsakanika L. A. Pavlopoulos C. Papadopoulou K. Ochsenkühn K. M. (2021). Study of microbial cultures for the bioleaching of scandium from alumina industry by-products. Metals 11 (6), 951. 10.3390/met11060951

[B109] Kiskira K. Lymperopoulou T. Lourentzatos I. Tsakanika L. A. Pavlopoulos C. Papadopoulou K. (2023). Bioleaching of scandium from bauxite residue using fungus *Aspergillus niger* . Waste Biomass Valorization 14 (10), 3377–3390. 10.1007/s12649-023-02116-5

[B110] Kobayashi K. Hattori T. Honda Y. Kirimura K. (2014). Oxalic acid production by citric acid-producing *Aspergillus niger* overexpressing the oxaloacetate hydrolase gene *oahA* . J. Industrial Microbiol. Biotechnol. 41 (5), 749–756. 10.1007/s10295-014-1419-2 24615146

[B111] Kriskova L. Ducman V. Loncnar M. Tesovnik A. Žibret G. Skentzou D. (2025). Alkali-activated mineral residues in construction: case studies on bauxite residue and steel slag pavement tiles. Materials 18 (2), 257. 10.3390/ma18020257 39859728 PMC11767172

[B112] Książek E. (2024). Citric acid: properties, microbial production, and applications in industries. Molecules 29 (1), 22. 10.3390/molecules29010022 38202605 PMC10779990

[B113] Kumar S. Kumar R. Bandopadhyay A. (2006). Innovative methodologies for the utilisation of wastes from metallurgical and allied industries. Resour. Conservation Recycl. 48 (4), 301–314. 10.1016/j.resconrec.2006.03.003

[B114] Kusumanjali G. Tejas S. Chatterjee A. Pasla D. (2025). Utilization of bauxite residue as fine aggregate for the development of structural concrete: an approach towards sustainable construction materials. Case Stud. Constr. Mater. 22, e04348. 10.1016/j.cscm.2025.e04348

[B115] Kvande H. (2015). “Occurrence and production of aluminum,” in Encyclopedia of inorganic and bioinorganic chemistry. Editor Scott R. A. 2 ed (Hoboken, NJ: John Wiley and Sons, Ltd), 1–10.

[B116] Kyrii S. Maletskyi Z. Klymenko N. Ratnaweera H. Mitchenko T. Dontsova T. (2023). Impact of modification by red mud components on the sorption properties of activated carbon. Appl. Surf. Sci. Adv. 16, 100412. 10.1016/j.apsadv.2023.100412

[B117] Lallemand C. Ambrosi J. P. Borschneck D. Angeletti B. Chaurand P. Campos A. (2022). Potential of ligand-promoted dissolution at mild pH for the selective recovery of rare earth elements in bauxite residues. ACS Sustain. Chem. and Eng. 10 (21), 6942–6951. 10.1021/acssuschemeng.1c08081

[B118] Li Z. Din J. Xu J. Liao C. Yin F. Lǚ T. (2013). Discovery of the REE minerals in the Wulong-Nanchuan bauxite deposits, Chongqing, China: insights on conditions of formation and processes. J. Geochem. Explor. 133, 88–102. 10.1016/j.gexplo.2013.06.016

[B119] Li Z. Bai T. Dai L. Wang F. Tao J. Meng S. (2016). A study of organic acid production in contrasts between two phosphate solubilizing fungi: *penicillium oxalicum* and *Aspergillus niger* . Sci. Rep. 6 (1), 25313. 10.1038/srep25313 27126606 PMC4850453

[B120] Li X. Liu M. Li W. Wang X. Wang S. Yin H. (2024). Toward sustainable utilization and production of tartaric acid. Chem. Rec. 24 (11), e202400099. 10.1002/tcr.202400099 39520318

[B121] Li Y. Wu B. Wen Y. Yang H. Jin L. Hu H. (2024). *In-situ* catalytic upgrading of lignin pyrolysis volatiles over red mud. J. Anal. Appl. Pyrolysis 181, 106599. 10.1016/j.jaap.2024.106599

[B122] Li S. Li L. Jiang Q. Wang J. Sun X. Zhang L. (2025). From glucose to green chemistry: breakthrough in microbial production of tartaric semialdehyde. Microb. Biotechnol. 18 (4), e70149. 10.1111/1751-7915.70149 40266016 PMC12016104

[B123] Liao J. Jiang J. Xue S. Qingyu C. Wu H. Manikandan R. (2018). A novel acid-producing fungus isolated from bauxite residue: the potential to reduce the alkalinity. Geomicrobiol. J. 35 (10), 840–847. 10.1080/01490451.2018.1479807

[B124] Liu R. Zhou H. (2022). Growth in ever-increasing acidity condition enhanced the adaptation and bioleaching ability of *Leptospirillum ferriphilum* . Int. Microbiol. 25 (3), 541–550. 10.1007/s10123-021-00227-4 35175436

[B125] Liu W. Yang J. Xiao B. (2009a). Application of Bayer red mud for iron recovery and building material production from alumosilicate residues. J. Hazard. Mater. 161 (1), 474–478. 10.1016/j.jhazmat.2008.03.122 18457916

[B126] Liu W. Yang J. Xiao B. (2009b). Review on treatment and utilization of bauxite residues in China. Int. J. Mineral Process. 93 (3), 220–231. 10.1016/j.minpro.2009.08.005

[B127] Liu W. Chen X. Li W. Yu Y. Yan K. (2014). Environmental assessment, management and utilization of red mud in China. J. Clean. Prod. 84, 606–610. 10.1016/j.jclepro.2014.06.080

[B128] Liu Y. Naidu R. Ming H. Dharmarajan R. Du J. (2016). Effects of thermal treatments on the characterisation and utilisation of red mud with sawdust additive. Waste Manag. Res. 34 (6), 518–526. 10.1177/0734242x16634197 26951343

[B129] Liu S. Guan X. Zhang S. Dou Z. Feng C. Zhang H. (2017). Sintered bayer red mud based ceramic bricks: microstructure evolution and alkalis immobilization mechanism. Ceram. Int. 43 (15), 13004–13008. 10.1016/j.ceramint.2017.07.036

[B130] Liu R. Chen Y. Tian Z. Mao Z. Cheng H. Zhou H. (2019). Enhancing microbial community performance on acid resistance by modified adaptive laboratory evolution. Bioresour. Technol. 287, 121416. 10.1016/j.biortech.2019.121416 31103940

[B131] Liu S. Liu Z. Zhu H. Wang Z. Guo J. Zhang X. (2023). The roles of red mud as desulfurization and denitrification in flue gas: a review. J. Environ. Chem. Eng. 11 (3), 109770. 10.1016/j.jece.2023.109770

[B132] Liu J. Pan X. Guo Y. Lv Z. Wei C. Yu H. (2024). Sustainable and efficient removal of phosphorus from wastewater through red mud residue after deep dealkalization. Colloids Surfaces A Physicochem. Eng. Aspects 700, 134782. 10.1016/j.colsurfa.2024.134782

[B133] Lotfy W. A. Ghanem K. M. El-Helow E. R. (2007). Citric acid production by a novel *Aspergillus niger* isolate: I. Mutagenesis and cost reduction studies. Bioresour. Technol. 98 (18), 3464–3469. 10.1016/j.biortech.2006.11.007 17223558

[B134] Lu F. Xiao T. Lin J. Ning Z. Long Q. Xiao L. (2017). Resources and extraction of gallium: a review. Hydrometallurgy 174, 105–115. 10.1016/j.hydromet.2017.10.010

[B135] Lu C. Wu S. Ma L. You F. Saha N. Bu H. (2025). Haloalkalitolerant plants drive alkaline mineral weathering and dealkalization of seawater-treated bauxite residue. Plant Soil 514 (2), 1993–2011. 10.1007/s11104-025-07501-8

[B136] Luo C. Liang P. Yang R. Gao J. Chen Q. Mo H. (2023). Mineralogical and geochemical constraints on the occurrence forms of REEs in Carboniferous karst bauxite, central Guizhou Province, southwest China: a case study of Lindai bauxite. Minerals 13 (3), 320. 10.3390/min13030320

[B137] Lyu F. Hu Y. Wang L. Sun W. (2021). Dealkalization processes of bauxite residue: a comprehensive review. J. Hazard. Mater. 403, 123671. 10.1016/j.jhazmat.2020.123671 33264875

[B138] Ma Y. Li B. Zhang X. Wang C. Chen W. (2022). Production of gluconic acid and its derivatives by microbial fermentation: process improvement based on integrated routes. Front. Bioeng. Biotechnol. 10, 864787. 10.3389/fbioe.2022.864787 35651548 PMC9149244

[B139] Mahgoub S. A. Kedra E. G. A. Abdelfattah H. I. Abdelbasit H. M. Alamoudi S. A. Al-Quwaie D. A. (2022). Bioconversion of some agro-residues into organic acids by cellulolytic rock-phosphate-solubilizing *Aspergillus japonicus* . Fermentation 8 (9), 437. 10.3390/fermentation8090437

[B140] Maluleke M. D. Kotsiopoulos A. Govender-Opitz E. Harrison S. T. L. (2024). Exploring microbial adaptation of immobilised acidophilic cultures to improve microbial oxidation rates and copper tolerance in e-waste bioleaching. Miner. Eng. 207, 108560. 10.1016/j.mineng.2023.108560

[B141] Mamlouk D. Gullo M. (2013). Acetic acid bacteria: physiology and carbon sources oxidation. Indian J. Microbiol. 53 (4), 377–384. 10.1007/s12088-013-0414-z 24426139 PMC3779290

[B142] Martins Y. J. C. Almeida A. C. M. Viegas B. M. do Nascimento R. A. Ribeiro N. F. P. (2020). Use of red mud from amazon region as an adsorbent for the removal of methylene blue: process optimization, isotherm and kinetic studies. Int. J. Environ. Sci. Technol. 17 (10), 4133–4148. 10.1007/s13762-020-02757-2

[B143] Matsushita K. Toyama H. Tonouchi N. Okamoto-Kainuma A. (2016). Acetic acid bacteria: ecology and physiology (Tokyo, Japan: Springer).

[B144] Matzapetakis M. Kourgiantakis M. Dakanali M. Raptopoulou C. P. Terzis A. Lakatos A. (2001). Synthesis, pH-dependent structural characterization, and solution behavior of aqueous aluminum and gallium citrate complexes. Inorg. Chem. 40 (8), 1734–1744. 10.1021/ic000461l 11312727

[B145] Mavrommati M. Daskalaki A. Papanikolaou S. Aggelis G. (2022). Adaptive laboratory evolution principles and applications in industrial biotechnology. Biotechnol. Adv. 54, 107795. 10.1016/j.biotechadv.2021.107795 34246744

[B146] Meng X. Zhao H. Zhao Y. Shen L. Gu G. Qiu G. (2023). Effective recovery of rare earth from (bio)leaching solution through precipitation of rare earth-citrate complex. Water Res. 233, 119752. 10.1016/j.watres.2023.119752 36812814

[B147] Merfort M. Herrmann U. Ha S. W. Elfari M. Bringer-Meyer S. Görisch H. (2006). Modification of the membrane‐bound glucose oxidation system in *Gluconobacter oxydans* significantly increases gluconate and 5‐keto‐D‐gluconic acid accumulation. Biotechnol. J. 1 (5), 556–563. 10.1002/biot.200600032 16892291

[B148] Merli G. Becci A. Amato A. Beolchini F. (2021). Acetic acid bioproduction: the technological innovation change. Sci. Total Environ. 798, 149292. 10.1016/j.scitotenv.2021.149292 34375263

[B149] Metilda D. L. Selvamony C. Anandakumar R. Seeni A. (2015). Investigations on optimum possibility of replacing cement partially by redmud in concrete. Sci. Res. Essays 10 (4), 137–143. 10.5897/sre2015.6166

[B150] Mizutani O. Masaki K. Gomi K. Iefuji H. (2012). Modified Cre-*loxP* recombination in *Aspergillus oryzae* by direct introduction of Cre recombinase for marker gene rescue. Appl. Environ. Microbiol. 78 (12), 4126–4133. 10.1128/aem.00080-12 22504800 PMC3370522

[B151] Morishita T. Yajima M. (1995). Incomplete operation of biosynthetic and bioenergetic functions of the citric acid cycle in multiple auxotrophic Lactobacilli. Biosci. Biotechnol. Biochem. 59 (2), 251–255. 10.1271/bbb.59.251

[B152] Murty C. V. G. K. Natarajan J. Rao N. J. (2023). in Beneficiation of mineral sands: a practical outlook. Editors Processing M. Rajendran S. Murty C. V. G. K. (Amsterdam, Netherlands: Elsevier), 167–220.

[B153] Naderi M. Shafaie S. Z. Karamoozian M. Gharanjik S. (2017). Optimization of parameters affecting recovery of copper from Sarcheshmeh low-grade sulfide ore using bioleaching. J. Min. Environ. 8 (4), 523–537. 10.22044/jme.2017.848

[B154] Nakano S. Fukaya M. Horinouchi S. (2006). Putative ABC transporter responsible for acetic acid resistance in *Acetobacter aceti* . Appl. Environ. Microbiol. 72 (1), 497–505. 10.1128/AEM.72.1.497-505.2006 16391084 PMC1352267

[B155] Narayanan R. P. Kazantzis N. K. Emmert M. H. (2019). Process for scandium recovery from Jamaican bauxite residue: a probabilistic economic assessment. Mater. Today Proc. 9, 578–586. 10.1016/j.matpr.2018.10.378

[B156] Naseri T. Beiki V. Mousavi S. M. Farnaud S. (2023). A comprehensive review of bioleaching optimization by statistical approaches: recycling mechanisms, factors affecting, challenges, and sustainability. RSC Adv. 13 (34), 23570–23589. 10.1039/d3ra03498d 37555097 PMC10404936

[B157] Nawab A. Yang X. Honaker R. (2022). Parametric study and speciation analysis of rare earth precipitation using oxalic acid in a chloride solution system. Miner. Eng. 176, 107352. 10.1016/j.mineng.2021.107352

[B158] Naykodi A. Patankar S. C. Thorat B. N. (2022). Alkaliphiles for comprehensive utilization of red mud (bauxite residue) - an alkaline waste from the alumina refinery. Environ. Sci. Pollut. Res. 30 (4), 9350–9368. 10.1007/s11356-022-24190-3 36480139

[B159] Nayl A. A. Arafa W. A. A. Abd-Elhamid A. I. Elkhashab R. A. (2020). Studying and spectral characterization for the separation of lanthanides from phosphate ore by organic and inorganic acids. J. Mater. Res. Technol. 9 (5), 10276–10290. 10.1016/j.jmrt.2020.07.007

[B160] Nishikawa R. Yoshida M. Noda T. Okuhara T. Taguchi G. Inatomi S. (2016). pFungiway: a series of plasmid vectors used for gene manipulation in fungi. Ann. Microbiol. 66 (2), 825–832. 10.1007/s13213-015-1166-2

[B161] Ochsenkühn-Petropulu M. Lyberopulu T. Ochsenkühn K. M. Parissakis G. (1996). Recovery of lanthanides and yttrium from red mud by selective leaching. Anal. Chim. Acta 319 (1), 249–254. 10.1016/0003-2670(95)00486-6

[B162] Oshoma C. E. Akor J. O. Ikhajiagbe B. Ikenebomeh M. J. (2022). Mutation of *Aspergillus* sp. using ultraviolet light and nitrous acid for amylase production from banana peels. Makara J. Sci. 26 (3), 209–216. 10.7454/mss.v26i3.1357

[B163] Padhan A. Paul B. (2025). Unlocking the potential of red mud: advanced strategies for economic optimization and sustainable recovery of critical minerals. J. Environ. Manag. 389, 126040. 10.1016/j.jenvman.2025.126040 40505561

[B164] Pathak A. Morrison L. Healy M. G. (2017). Catalytic potential of selected metal ions for bioleaching, and potential techno-economic and environmental issues: a critical review. Bioresour. Technol. 229, 211–221. 10.1016/j.biortech.2017.01.001 28108075

[B165] Pathak A. Kothari R. Vinoba M. Habibi N. Tyagi V. V. (2021). Fungal bioleaching of metals from refinery spent catalysts: a critical review of current research, challenges, and future directions. J. Environ. Manag. 280, 111789. 10.1016/j.jenvman.2020.111789 33370668

[B166] Patterson S. H. Kurtz H. F. Olson J. C. Neeley C. L. (1986). World bauxite resources. Washington, DC: U.S. Geological Survey.

[B167] Patyshakuliyeva A. Arentshorst M. Allijn I. E. Ram A. F. J. de Vries R. P. Gelber I. B. (2016). Improving cellulase production by *Aspergillus niger* using adaptive evolution. Biotechnol. Lett. 38 (6), 969–974. 10.1007/s10529-016-2060-0 26879082 PMC4853455

[B168] Pedram H. Hosseini M. R. Bahrami A. (2020). Utilization of *A. niger* strains isolated from pistachio husk and grape skin in the bioleaching of valuable elements from red mud. Hydrometallurgy 198, 105495. 10.1016/j.hydromet.2020.105495

[B169] Pilla G. Hertel T. Blanpain B. Pontikes Y. (2025). A sustainable approach for concurrent recovery of metals from H_2_ reduced bauxite residue (“red mud”): process optimization. Resour. Conservation Recycl. 215, 108051. 10.1016/j.resconrec.2024.108051

[B170] Portnoy V. A. Bezdan D. Zengler K. (2011). Adaptive laboratory evolution - harnessing the power of biology for metabolic engineering. Curr. Opin. Biotechnol. 22 (4), 590–594. 10.1016/j.copbio.2011.03.007 21497080

[B171] Pott R. Johnstone-Robertson M. Verster B. Rumjeet S. Nkadimeng L. Raper T. (2018). “Wastewater biorefineries: integrating water treatment and value recovery,” in The nexus: energy, environment and climate change. Editors Leal Filho W. Surroop D. (Cham, Switzerland: Springer), 289–302.

[B172] Pozdnyakov I. P. Wu F. Melnikov A. A. Grivin V. P. Bazhin N. M. Chekalin S. V. (2013). Photochemistry of iron(iii)-lactic acid complex in aqueous solutions. Russ. Chem. Bull. 62 (7), 1579–1585. 10.1007/s11172-013-0227-6

[B173] Prasad P. M. Singh M. (1997). Problems in the disposal and utilization of red muds. Banaras Metallurgist 14-15, 127–140.

[B174] Pratap B. (2024). Analysis of mechanical properties of fly ash- and bauxite residue-based geopolymer concrete using ANN, random forest and counter-propagation neural network. Asian J. Civ. Eng. 25 (5), 4303–4317. 10.1007/s42107-024-01049-1

[B175] Prodius D. Klocke M. Smetana V. Alammar T. Perez Garcia M. Windus T. L. (2020). Rationally designed rare earth separation by selective oxalate solubilization. Chem. Commun. 56 (77), 11386–11389. 10.1039/d0cc02270e 32894275

[B176] Promentilla M. A. B. Thang N. H. Kien P. T. Hinode H. Bacani F. T. Gallardo S. M. (2016). Optimizing ternary-blended geopolymers with multi-response surface analysis. Waste Biomass Valorization 7 (4), 929–939. 10.1007/s12649-016-9490-8

[B177] Qi Z. Wang W. Yang H. Xia X. Yu X. (2014). Mutation of *Acetobacter pasteurianus* by UV irradiation under acidic stress for high‐acidity vinegar fermentation. Int. J. Food Sci. and Technol. 49 (2), 468–476. 10.1111/ijfs.12324

[B178] Qu Y. Lian B. (2013). Bioleaching of rare earth and radioactive elements from red mud using *Penicillium tricolor* RM-10. Bioresour. Technol. 136, 16–23. 10.1016/j.biortech.2013.03.070 23548400

[B179] Qu Y. Lian B. Mo B. Liu C. (2013). Bioleaching of heavy metals from red mud using *Aspergillus niger* . Hydrometallurgy 136, 71–77. 10.1016/j.hydromet.2013.03.006

[B180] Qu Y. Li H. Tian W. Wang X. Wang X. Jia X. (2015). Leaching of valuable metals from red mud via batch and continuous processes by using fungi. Miner. Eng. 81, 1–4. 10.1016/j.mineng.2015.07.022

[B181] Qu Y. Li H. Wang X. Tian W. Shi B. Yao M. (2019a). Selective parameters and bioleaching kinetics for leaching vanadium from red mud using *Aspergillus niger* and *Penicillium tricolor* . Minerals 9 (11), 697. 10.3390/min9110697

[B182] Qu Y. Li H. Wang X. Tian W. Shi B. Yao M. (2019b). Bioleaching of major, rare earth, and radioactive elements from red mud by using indigenous chemoheterotrophic bacterium *Acetobacter* sp. Minerals 9 (2), 67. 10.3390/min9020067

[B183] Qu Y. Li H. Shi B. Gu H. Yan G. Liu Z. (2022). Bioleaching performance of titanium from bauxite residue under a continuous mode using *Penicillium Tricolor* . Bull. Environ. Contam. Toxicol. 109 (1), 61–67. 10.1007/s00128-022-03518-2 35412056

[B184] Rai S. Wasewar K. L. Agnihotri A. (2017). Treatment of alumina refinery waste (red mud) through neutralization techniques: a review. Waste Manag. Res. 35 (6), 563–580. 10.1177/0734242X17696147 28566030

[B185] Raj R. Yadav B. Yadav J. S. Kumar S. (2024). Red mud utilisation for sustainable construction and soil improvement: a comprehensive review. Discov. Sustain. 5 (1), 398. 10.1007/s43621-024-00619-2

[B186] Rastegar S. O. Samadi A. Ahmadnezhad P. Nazari T. (2024). Bioleaching of sewage sludge for copper extraction using *Acidithiobacillus thiooxidans*: optimization and ecological risk assessment. Chemosphere 353, 141466. 10.1016/j.chemosphere.2024.141466 38364921

[B187] Ren W. X. Li P. J. Geng Y. Li X. J. (2009). Biological leaching of heavy metals from a contaminated soil by *Aspergillus niger* . J. Hazard. Mater. 167 (1), 164–169. 10.1016/j.jhazmat.2008.12.104 19232463

[B188] Rivera R. M. Ounoughene G. Borra C. R. Binnemans K. Van Gerven T. (2017). Neutralisation of bauxite residue by carbon dioxide prior to acidic leaching for metal recovery. Miner. Eng. 112, 92–102. 10.1016/j.mineng.2017.07.011

[B189] Rivera R. M. Ounoughene G. Malfliet A. Vind J. Panias D. Vassiliadou V. (2019). A study of the occurrence of selected rare-earth elements in neutralized-leached bauxite residue and comparison with untreated bauxite residue. J. Sustain. Metallurgy 5 (1), 57–68. 10.1007/s40831-018-0206-0

[B190] Rus A. M. M. Winarko R. Chaerun S. K. Mufakhir F. R. Astuti W. Minwal W. P. (2024). Bioleaching of rare earth elements (REEs) from Indonesian red mud by the bacterium *Bacillus nitratireducens* strain SKC/L-2. E3S Web Conf. 543, 02014. 10.1051/e3sconf/202454302014

[B191] Rushendra Revathy T. D. Ramachandran A. Palanivelu K. (2021). Sequestration of CO_2_ by red mud with flue gas using response surface methodology. Carbon Manag. 12 (2), 139–151. 10.1080/17583004.2021.1893127

[B192] Saldaña M. Jeldres M. Galleguillos Madrid F. M. Gallegos S. Salazar I. Robles P. (2023). Bioleaching modeling - a review. Materials 16 (10), 3812. 10.3390/ma16103812 37241440 PMC10224567

[B193] Salim M. U. Mosaberpanah M. A. Danish A. Ahmad N. Khalid R. A. Moro C. (2023). Role of bauxite residue as a binding material and its effect on engineering properties of cementitious composites: a review. Constr. Build. Mater. 409, 133844. 10.1016/j.conbuildmat.2023.133844

[B194] Santini T. C. Fey M. V. (2016). Assessment of technosol formation and *in situ* remediation in capped alkaline tailings. CATENA 136, 17–29. 10.1016/j.catena.2015.08.006

[B195] Santini T. C. Kerr J. L. Warren L. A. (2015). Microbially-driven strategies for bioremediation of bauxite residue. J. Hazard. Mater. 293, 131–157. 10.1016/j.jhazmat.2015.03.024 25867516

[B196] Sarma J. Sengupta A. Laskar M. K. Sengupta S. Tenguria S. Kumar A. (2023). “Microbial adaptations in extreme environmental conditions,” in Bacterial survival in the hostile environment. Editors Kumar A. Tenguria S. (London, UK: Academic Press), 193–206.

[B197] Sawant O. Mahale S. Ramchandran V. Nagaraj G. Bankar A. (2018). Fungal citric acid production using waste materials: a mini-review. J. Microbiol. Biotechnol. Food Sci. 8 (2), 821–828. 10.15414/jmbfs.2018.8.2.821-828

[B198] Schmitz A. M. Pian B. Marecos S. Wu M. Holycross M. Gazel E. (2025). High efficiency rare earth element bioleaching with systems biology guided engineering of *Gluconobacter oxydans* . Commun. Biol. 8 (1), 815. 10.1038/s42003-025-08109-5 40425722 PMC12117071

[B199] Shah S. S. Palmieri M. C. Sponchiado S. R. P. Bevilaqua D. (2020). Enhanced bio-recovery of aluminum from low-grade bauxite using adapted fungal strains. Braz. J. Microbiol. 51 (4), 1909–1918. 10.1007/s42770-020-00342-w 32748245 PMC7688833

[B200] Shah S. S. Palmieri M. C. Sponchiado S. R. P. Bevilaqua D. (2022). A sustainable approach on biomining of low-grade bauxite by *P. simplicissimum* using molasses medium. Braz. J. Microbiol. 53 (2), 831–843. 10.1007/s42770-022-00683-8 35079978 PMC9151954

[B201] Shen Q. Ruan H. Zhang H. Wu T. Zhu K. Han W. (2024). Utilization of CRISPR-Cas genome editing technology in filamentous fungi: function and advancement potentiality. Front. Microbiol. 15, 1375120. 10.3389/fmicb.2024.1375120 38605715 PMC11007153

[B202] Shoppert A. Valeev D. Diallo M. M. Loginova I. Beavogui M. C. Rakhmonov A. (2022). High-iron bauxite residue (red mud) valorization using hydrochemical conversion of goethite to magnetite. Materials 15 (23), 8423. 10.3390/ma15238423 36499918 PMC9741149

[B203] Silveira L. M. V. Cincotto M. A. Romano R. C. O. Pileggi R. G. (2025). Early-age properties of Portland cement with untreated bauxite residue (UBR) as SCM for large-scale adoption. Constr. Build. Mater. 464, 140139. 10.1016/j.conbuildmat.2025.140139

[B204] Smičiklas I. Jović M. Janković M. Smiljanić S. Onjia A. (2021). Environmental safety aspects of solid residues resulting from acid mine drainage neutralization with fresh and aged red mud. Water, Air, and Soil Pollut. 232 (12), 490. 10.1007/s11270-021-05442-3

[B205] Smith P. (2017). Reactions of lime under high temperature bayer digestion conditions. Hydrometallurgy 170, 16–23. 10.1016/j.hydromet.2016.02.011

[B206] Smith R. M. Martell A. E. Motekaitis R. J. (2004). NIST critically selected stability constants of metal complexes database.

[B207] Smith Stegen K. (2015). Heavy rare earths, permanent magnets, and renewable energies: an imminent crisis. Energy Policy 79, 1–8. 10.1016/j.enpol.2014.12.015

[B208] Snars K. Gilkes R. J. (2009). Evaluation of bauxite residues (red muds) of different origins for environmental applications. Appl. Clay Sci. 46 (1), 13–20. 10.1016/j.clay.2009.06.014

[B209] Steiger M. G. Rassinger A. Mattanovich D. Sauer M. (2019). Engineering of the citrate exporter protein enables high citric acid production in *Aspergillus niger* . Metab. Eng. 52, 224–231. 10.1016/j.ymben.2018.12.004 30553933

[B210] Stopić S. Damjanović V. Filipović R. Kamara M. Friedrich B. (2023). Treatment of bauxite residues: acidic leaching (first part). Vojnoteh. Glas. 71 (4), 1069–1086. 10.5937/vojtehg71-46212

[B211] Šulc J. Jelínková H. (2013). “Solid-state lasers for medical applications,” in Lasers for medical applications: diagnostics, therapy and surgery. Editor Jelínková H. (Cambridge, UK: Woodhead Publishing), 127–176.

[B212] Sun C. Chen J. Tian K. Peng D. Liao X. Wu X. (2019). Geochemical characteristics and toxic elements in alumina refining wastes and leachates from management facilities. Int. J. Environ. Res. Public Health 16 (7), 1297. 10.3390/ijerph16071297 30978989 PMC6480639

[B213] Suresh G. Ramesh M. R. Srinath M. S. (2023). “Surface engineered titanium alloys for biomedical, automotive, and aerospace applications,” in Advances in processing of lightweight metal alloys and composites. Editors Vignesh R. V. Padmanaban R. Govindaraju M. (Singapore: Springer), 89–102.

[B216] Sushil S. Batra V. S. (2008). Catalytic applications of red mud, an aluminium industry waste: a review. Appl. Catal. B Environ. 81 (1), 64–77. 10.1016/j.apcatb.2007.12.002

[B217] Sushil S. Batra V. S. (2012). Modification of red mud by acid treatment and its application for CO removal. J. Hazard. Mater. 203-204, 264–273. 10.1016/j.jhazmat.2011.12.007 22204836

[B218] Swain B. Akcil A. Lee J. C. (2022). Red mud valorization an industrial waste circular economy challenge; review over processes and their chemistry. Crit. Rev. Environ. Sci. Technol. 52 (4), 520–570. 10.1080/10643389.2020.1829898

[B219] Tanvar H. Mishra B. (2025). Environmental management by recycling of bauxite residue. J. Adv. Manuf. Process. 7 (3), e70010. 10.1002/amp2.70010

[B220] Tao L. Wu H. Wang J. Li B. Wang X. Q. Ning P. (2019). Removal of SO_2_ from flue gas using bayer red mud: influence factors and mechanism. J. Central South Univ. 26 (2), 467–478. 10.1007/s11771-019-4019-5

[B221] Thomas J. B. (2018). “Titanium,” in Encyclopedia of geochemistry. Editor White W. M. (Cham, Switzerland: Springer), 1445–1451.

[B222] Thompson V. S. Gupta M. Jin H. Vahidi E. Yim M. Jindra M. A. (2017). Techno-economic and life cycle analysis for bioleaching rare-earth elements from waste materials. ACS Sustain. Chem. and Eng. 6 (2), 1602–1609. 10.1021/acssuschemeng.7b02771

[B223] Thygesen P. (2019). Clarifying the regulation of genome editing in Australia: situation for genetically modified organisms. Transgenic Res. 28 (2), 151–159. 10.1007/s11248-019-00151-4 31321698

[B224] Thygesen P. (2024). Regulation of genome edited organisms in Australia. Transgenic Res. 33 (6), 545–550. 10.1007/s11248-024-00411-y 39446213

[B225] Toli A. Kotsanis D. Psoma M. Marinos D. Davris P. Balomenos E. (2023). The efficient use of sulfuric acid in bauxite residue leaching. Mater. Proc. 15 (1), 53. 10.3390/materproc2023015053

[B226] Trivedi A. Hait S. (2024). Fungal bioleaching of metals from WPCBs of mobile phones employing mixed *Aspergillus* spp.: optimization and predictive modelling by RSM and AI models. J. Environ. Manag. 349, 119565. 10.1016/j.jenvman.2023.119565 37976642

[B227] Tsaramyrsi M. Kavousanaki D. Raptopoulou C. P. Terzis A. Salifoglou A. (2001). Systematic synthesis, structural characterization, and reactivity studies of vanadium(V)-citrate anions [VO_2_(C_6_H_6_O_7_)]_2_ ^2-^, isolated from aqueous solutions in the presence of different cations. Inorganica Chim. Acta 320 (1), 47–59. 10.1016/S0020-1693(01)00464-9

[B228] U. S. Environmental Protection Agency (2024). TENORM: bauxite and alumina production wastes. Available online at: https://www.epa.gov/radiation/tenorm-bauxite-and-alumina-production-wastes (Accessed July 20, 2025).

[B229] Ujaczki É. Zimmermann Y. S. Gasser C. A. Molnár M. Feigl V. Lenz M. (2017). Red mud as secondary source for critical raw materials - extraction study. J. Chem. Technol. and Biotechnol. 92 (11), 2835–2844. 10.1002/jctb.5300

[B230] United Nations General Assembly (2015). Transforming our world: the 2030 agenda for sustainable development, A/RES/70/1. Available online at: https://www.refworld.org/legal/resolution/unga/2015/en/111816 (Accessed July 20, 2025).

[B214] U.S. Geological Survey (2024). Mineral commodity summaries 2024. Reston, VA: U.S. Geological Survey. 10.3133/mcs2024

[B215] U.S. Geological Survey (2025). Mineral commodity summaries 2025. Reston, VA: U.S. Geological Survey. 10.3133/mcs2025

[B231] Vakilchap F. Mousavi S. M. Shojaosadati S. A. (2016). Role of *Aspergillus niger* in recovery enhancement of valuable metals from produced red mud in Bayer process. Bioresour. Technol. 218, 991–998. 10.1016/j.biortech.2016.07.059 27450129

[B232] Valix M. Loon L. O. (2003). Adaptive tolerance behaviour of fungi in heavy metals. Miner. Eng. 16 (3), 193–198. 10.1016/s0892-6875(03)00004-9

[B233] Van Wyk N. Fischer D. Wilbers D. Harrison S. T. L. Kotsiopoulos A. Dopson M. (2024). Toward the bioleaching of bauxite residue by *Gluconobacter oxydans* . J. Appl. Microbiol. 135 (11), lxae279. 10.1093/jambio/lxae279 39501498

[B234] Venturini-Soriano M. Berthon G. (2001). Aluminum speciation studies in biological fluids. J. Inorg. Biochem. 85 (2), 143–154. 10.1016/s0162-0134(01)00206-9 11410234

[B235] Vielma C. A. Svobodova-Sedlackova A. Chimenos J. M. Fernández A. I. Berlanga C. Rodriguez R. (2025). Valorisation of red mud: disclosing the potential of an abundant residue. Sustainability 17 (5), 1849. 10.3390/su17051849

[B236] Villa Gomez D. Whitworth A. J. Vaughan J. Sultana U. Ledezma P. Parbhakar-Fox A. (2024). Review on developments in technologies for critical metal recovery from mining and processing wastes. Mineral Process. Extr. Metallurgy Rev. 46 (7), 751–770. 10.1080/08827508.2024.2408015

[B237] Vind J. Alexandri A. Vassiliadou V. Panias D. (2018a). Distribution of selected trace elements in the Bayer process. Metals 8 (5), 327. 10.3390/met8050327

[B238] Vind J. Malfliet A. Blanpain B. Tsakiridis P. E. Tkaczyk A. H. Vassiliadou V. (2018b). Rare earth element phases in bauxite residue. Minerals 8 (2), 77. 10.3390/min8020077

[B239] Vind J. Malfliet A. Bonomi C. Paiste P. Sajó I. E. Blanpain B. (2018c). Modes of occurrences of scandium in Greek bauxite and bauxite residue. Miner. Eng. 123, 35–48. 10.1016/j.mineng.2018.04.025

[B240] Visual MINTEQ (2021). Visual MINTEQ - a freeware chemical equilibrium model for the calculation of metal speciation, solubility equilibria, sorption etc. for natural waters. Available online at: https://vminteq.com/ (Accessed July 20, 2025).

[B241] Viyasun K. Anuradha R. Thangapandi K. Kumar D. S. Sivakrishna A. Gobinath R. (2021). Investigation on performance of red mud based concrete. Mater. Today Proc. 39, 796–799. 10.1016/j.matpr.2020.09.637

[B242] Vu V. H. Pham T. A. Kim K. (2009). Fungal strain improvement for cellulase production using repeated and sequential mutagenesis. Mycobiology 37 (4), 267–271. 10.4489/myco.2009.37.4.267 23983546 PMC3749416

[B243] Vukosav P. Tomišić V. Mlakar M. (2010). Iron(III)‐complexes engaged in the biochemical processes in seawater. II. Voltammetry of Fe(III)‐malate complexes in model aqueous solution. Electroanalysis 22 (19), 2179–2186. 10.1002/elan.200900632

[B244] Vukosav P. Mlakar M. Tomišić V. (2012). Revision of iron(III)-citrate speciation in aqueous solution. Voltammetric and spectrophotometric studies. Anal. Chim. Acta 745, 85–91. 10.1016/j.aca.2012.07.036 22938610

[B245] Wang P. Liu D. Y. (2012). Physical and chemical properties of sintering red mud and Bayer red mud and the implications for beneficial utilization. Materials 5 (10), 1800–1810. 10.3390/ma5101800

[B246] Wang S. Li Z. Bai X. Yi W. Fu P. (2018). Catalytic pyrolysis of lignin with red mud derived hierarchical porous catalyst for alkyl-phenols and hydrocarbons production. J. Anal. Appl. Pyrolysis 136, 8–17. 10.1016/j.jaap.2018.10.024

[B247] Wang Y. Li Z. Li Z. Bao X. (2019). Over-expression of Bgl1 from *Aspergillus niger* in *Penicillium oxalicum* . AIP Conf. Proc. 2110 (1), 020027. 10.1063/1.5110821

[B248] Wen Y. Liu P. Wang Q. Zhao S. Tang Y. (2024). Organic ligand-mediated dissolution and fractionation of rare-earth elements (REEs) from carbonate and phosphate minerals. ACS Earth Space Chem. 8 (5), 1048–1061. 10.1021/acsearthspacechem.4c00009 38774356 PMC11103772

[B249] Wozniak C. A. McClung G. Gagliardi J. Segal M. Matthews K. (2012). “Regulation of genetically engineered microorganisms under FIFRA, FFDCA,” in Regulation of agricultural biotechnology: the United States and Canada. Editors Wozniak C. A. McHughen A. (Dordrecht, Netherlands: Springer), 57–94.

[B250] Wu X. Yao H. Cao L. Zheng Z. Chen X. Zhang M. (2017). Improving acetic acid production by over-expressing PQQ-ADH in *Acetobacter pasteurianus* . Front. Microbiol. 8, 1713. 10.3389/fmicb.2017.01713 28932219 PMC5592214

[B251] Wu T. Li J. Tian C. (2023). Fungal carboxylate transporters: recent manipulations and applications. Appl. Microbiol. Biotechnol. 107 (19), 5909–5922. 10.1007/s00253-023-12720-z 37561180

[B252] Xia C. Griffith W. (2018). Direct oxalate precipitation for rare earth elements recovery. Can. Pat. Appl. No PCT/CA2017/050508.

[B253] Xu Y. Shan L. Zhou Y. Xie Z. Ball A. S. Cao W. (2019). Development of a Cre-*loxP*-based genetic system in *Aspergillus niger* ATCC1015 and its application to construction of efficient organic acid-producing cell factories. Appl. Microbiol. Biotechnol. 103 (19), 8105–8114. 10.1007/s00253-019-10054-3 31392377

[B254] Xu Y. Zhou Y. Cao W. Liu H. (2020). Improved production of malic acid in *Aspergillus niger* by abolishing citric acid accumulation and enhancing glycolytic flux. ACS Synth. Biol. 9 (6), 1418–1425. 10.1021/acssynbio.0c00096 32379964

[B255] Xu H. Li D. Qian A. Jiang L. Cai J. Huang L. (2024). Red mud as the catalyst for energy and environmental catalysis: a review. Energy and Fuels 38 (15), 13737–13759. 10.1021/acs.energyfuels.4c02269

[B256] Xue S. Kong X. Zhu F. Hartley W. Li X. Li Y. (2016). Proposal for management and alkalinity transformation of bauxite residue in China. Environ. Sci. Pollut. Res. 23 (13), 12822–12834. 10.1007/s11356-016-6478-7 27023808

[B257] Xue X. Bi F. Liu B. Li J. Zhang L. Zhang J. (2021). Improving citric acid production of an industrial *Aspergillus niger* CGMCC 10142: identification and overexpression of a high-affinity glucose transporter with different promoters. Microb. Cell Factories 20 (1), 168. 10.1186/s12934-021-01659-3 34446025 PMC8394697

[B258] Yadav P. Chauhan A. K. Singh R. B. Khan S. Halabi G. (2022). “Organic acids: microbial sources, production, and applications,” in Functional foods and nutraceuticals in metabolic and non-communicable diseases. Editors Singh R. B. Watanabe S. Isaza A. A. (London, UK: Academic Press), 325–337.

[B259] Yang J. Wang Q. Wang Q. Wu T. (2008a). Comparisons of one-step and two-step bioleaching for heavy metals removed from municipal solid waste incineration fly ash. Environ. Eng. Sci. 25 (5), 783–789. 10.1089/ees.2007.0211

[B260] Yang J. Zhang D. Hou J. He B. Xiao B. (2008b). Preparation of glass-ceramics from red mud in the aluminium industries. Ceram. Int. 34 (1), 125–130. 10.1016/j.ceramint.2006.08.013

[B261] Yang T. Wang Y. Sheng L. He C. Sun W. He Q. (2020). Enhancing Cd(II) sorption by red mud with heat treatment: performance and mechanisms of sorption. J. Environ. Manag. 255, 109866. 10.1016/j.jenvman.2019.109866 31759202

[B262] Yang S. Li K. Liu H. Lu J. Yang H. Wu D. (2024). Enhancing citric acid tolerance of *Acetobacter tropicalis* using chemical and physical mutagenesis and adaptive evolution to improve the quality of lemon fruit vinegar. J. Food Sci. 89 (5), 2581–2596. 10.1111/1750-3841.17031 38551187

[B263] Yang F. Zhang J. Xie M. Cui W. Dong X. (2025). Evaluation of compression index of red mud by machine learning interpretability methods. Comput. Geotechnics 181, 107130. 10.1016/j.compgeo.2025.107130

[B264] Yang J. Liu X. Cui K. Lyu J. Liu H. Qiu J. (2025). Hazards and dealkalization technology of red mud - a critical review. Minerals 15 (4), 343. 10.3390/min15040343

[B265] Yi X. Lu Y. He G. (2024). Aluminum demand and low carbon development scenarios for major countries by 2050. J. Clean. Prod. 475, 143647. 10.1016/j.jclepro.2024.143647

[B266] Yıldız T. D. Tombal-Kara T. D. Kurşun-Ünver İ. (2024). “Challenges and recovery opportunities in waste management during the mining and enrichment processes of rare earth element-containing ores,” in Trash or treasure: entrepreneurial opportunities in waste management. Editors Singh P. Borthakur A. (Cham, Switzerland: Springer), 277–306.

[B267] Yoshioka I. Kobayashi K. Kirimura K. (2020). Overexpression of the gene encoding alternative oxidase for enhanced glucose consumption in oxalic acid producing *Aspergillus niger* expressing oxaloacetate hydrolase gene. J. Biosci. Bioeng. 129 (2), 172–176. 10.1016/j.jbiosc.2019.08.014 31611058

[B268] Yuxin L. Wenjie Z. Congzhong T. Wentao H. Zhijun Z. Zhou F. (2025). A critical review of gallium production: resources and extraction technologies. Miner. Eng. 228, 109320. 10.1016/j.mineng.2025.109320

[B269] Zabiszak M. Frymark J. Grajewski J. Jastrzab R. (2024). Spectroscopic studies of lanthanide(III) complexes with L-malic acid in binary systems. Int. J. Mol. Sci. 25 (17), 9210. 10.3390/ijms25179210 39273158 PMC11395662

[B270] Zapp P. Schreiber A. Marx J. Kuckshinrichs W. (2022). Environmental impacts of rare earth production. MRS Bull. 47 (3), 267–275. 10.1557/s43577-022-00286-6 35316936 PMC8929459

[B271] Zhang D. X. Lu H. L. Liao X. St. Leger R. J. Nuss D. L. (2013). Simple and efficient recycling of fungal selectable marker genes with the Cre-*loxP* recombination system via anastomosis. Fungal Genet. Biol. 61, 1–8. 10.1016/j.fgb.2013.08.013 24007936 PMC3838493

[B272] Zhang D. Chen H. Nie Z. Y. Xia J. Li E. Fan X. (2020). Extraction of Al and rare earths (Ce, Gd, Sc, Y) from red mud by aerobic and anaerobic bi-stage bioleaching. Chem. Eng. J. 401, 125914. 10.1016/j.cej.2020.125914

[B273] Zhang R. Zhang Z. Wu J. Wang L. (2022). Spatial characteristics and risk assessment of heavy metals in the soil-vegetation system of a red mud slag yard, SW China. Bull. Environ. Contam. Toxicol. 109 (1), 122–129. 10.1007/s00128-022-03493-8 35244751

[B274] Zhang C. Shi M. Xu Y. Yang D. Lu L. Xue F. (2024). Conditional expression of FumA in *Aspergillus niger* enhances synthesis of L-malic acid. Appl. Environ. Microbiol. 90 (4), e00008-24. 10.1128/aem.00008-24 38506527 PMC11022578

[B275] Zhao S. Tan M. Z. Wang R. X. Ye F. T. Chen Y. P. Luo X. M. (2022). Combination of genetic engineering and random mutagenesis for improving production of raw-starch-degrading enzymes in *Penicillium oxalicum* . Microb. Cell Factories 21 (1), 272. 10.1186/s12934-022-01997-w 36566178 PMC9790131

[B276] Zhou X. Xu Y. (2019). Integrative process for sugarcane bagasse biorefinery to co-produce xylooligosaccharides and gluconic acid. Bioresour. Technol. 282, 81–87. 10.1016/j.biortech.2019.02.129 30852335

[B277] Zotta T. Parente E. Ricciardi A. (2017). Aerobic metabolism in the genus *Lactobacillus*: impact on stress response and potential applications in the food industry. J. Appl. Microbiol. 122 (4), 857–869. 10.1111/jam.13399 28063197

