# Supplementary material for: Bioleaching for critical metal recovery from bauxite residue- unlocking waste valorization
Source: Front Bioeng Biotechnol. 2025 Nov 26;13:1685819. doi: 10.3389/fbioe.2025.1685819 (PMC12689584; doi:10.3389/fbioe.2025.1685819)
Supplement: Supplementary file 1 [file DataSheet1.docx]

Supplementary Material: Bioleaching for Critical Metal Recovery from Bauxite Residue- Unlocking Waste Valorization

# Earth’s crust average concentration used for Figure 2.

Table 1: Average earth's crust concentration. Adapted from (Thomas, 2018; Geoscience Australia, 2024)

|  | Element | Earth's crust abundance | Unit |
| --- | --- | --- | --- |
| LREEs | La | 20 | ppm |
|  | Ce | 43 | ppm |
|  | Pr | 4.9 | ppm |
|  | Nd | 20 | ppm |
|  | Pm |  | ppm |
|  | Sm | 3.9 | ppm |
|  | Eu | 1.1 | ppm |
|  | Gd | 3.7 | ppm |
| HREEs | Tb | 0.6 | ppm |
|  | Dy | 3.6 | ppm |
|  | Ho | 0.77 | ppm |
|  | Er | 2.1 | ppm |
|  | Tm | 0.28 | ppm |
|  | Yb | 1.9 | ppm |
|  | Lu | 0.3 | ppm |
|  | Y | 31 | ppm |
|  | Sc | 22 | ppm |
| Other CM | Ga | 16 | ppm |
|  | V | 138 | ppm |
|  | Ti | 0.52 | % wt |

# References

Geoscience Australia (2024). *Critical minerals and their uses* [Online]. Available: <https://www.ga.gov.au/scientific-topics/minerals/critical-minerals/critical-minerals-and-their-uses> [Accessed July 20, 2025].

Thomas, J.B. (2018). "Titanium," in Encyclopedia of Geochemistry*,* ed. W.M. White. (Cham, Switzerland: Springer), 1445-1451.
